# Methodology of murine lung cancer mimics clinical lung adenocarcinoma progression and metastasis

**DOI:** 10.1038/s41598-025-90344-1

**Published:** 2025-02-28

**Authors:** Edison Q. Kim, Emily Y. Kim, Eric P. Knott, Yujie Wang, Cheng-Bang Chen, Jose R. Conejo-Garcia, Medhi Wangpaichitr, Diane C. Lim

**Affiliations:** 1 Research Services, Miami VA Healthcare System, Miami, FL 33125 USA; 2 South Florida Veterans Affairs Foundation for Research and Education, Miami, FL 33125 USA; 3 Department of Industrial and Systems Engineering, University of Miami, Miami, FL 33146 USA; 4 Department of Integrative Immunobiology, Duke University School of Medicine, Durham, NC 27710 USA; 5 Department of Surgery, Cardiothoracic Surgery, University of Miami, Miami, FL 33136 USA; 6 Division of Pulmonary/Critical Care/Sleep, University of Miami, Miami, FL 33136 USA; 7 Division of Sleep Medicine, Miami VA Healthcare System, Miami, FL 33125 USA

**Keywords:** Non-small-cell lung cancer, Metastasis, Cancer models

## Abstract

Lung cancer is the leading cause of cancer-related deaths, of which adenocarcinoma is the most common subtype. Despite this, lung adenocarcinoma and its metastasis are poorly understood, due to difficulties in feasibly recapitulating disease progression and predicting clinical benefits of therapy. We outline a methodology to develop immunogenic orthotopic lung adenocarcinoma mouse models, by injecting cell-specific cre viruses into the lung of a genetically engineered mouse, which mirrors cancer progression defined by the International Association for the Study of Lung Cancer. Evaluation of different cre virus/concentrations models demonstrate remarkable consistency in cancer initiation and metastasis, allowing for high throughput, while showing differences in timing and severity, offering greater flexibility when selecting models. Histological and immune profiles reflect clinical observations suggesting similar mechanisms are recapitulated and preliminary data show resultant tumors to be responsive to clinical treatments. We present a clinically relevant, next-generation murine model for studying lung adenocarcinoma.

**Supplementary Information:**

The online version contains supplementary material available at 10.1038/s41598-025-90344-1.

## Introduction

The International Association for the Study of Lung Cancer (IASLC) and World Health Organization have revised the lung cancer Tumor-Node-Metastasis (TNM) classification system now in the 8th edition, correlating stages to patient survival^1,2^. Current 5-year survival for early Stage I ranges from 51%^3^ to 70%,^4^ compared to late Stage IIIB at 10–30%^5–8^ and Stage IV at 5–10%^8–10^ with ~ 80% of lung cancer deaths attributed to metastasis^11^. In addition to TNM classification, the IASLC uses histology to classify lung cancer and has been used for decades to provide prognoses and treatment plans. The most common histological subtype, adenocarcinoma, accounts for 50% of all lung cancers^12^. While earlier stages have better patient survival, and lung cancer screenings have significantly increased early stage detection^13^, the majority of patients still present at a late stage when curative surgery is not offered^14–16^, resulting in lung cancer still causing the most cancer-related deaths^17^. Therefore, to improve overall survival, there has been calls for mouse models to better reflect clinical lung cancer progression and response to therapy^18^.

Orthotopic mouse models of lung cancer are essential for more clinically relevant results due to the lung’s unique anatomy of ventilation and perfusion^19^ having a profound effect on Tumor Microenvironment (TME). Genetically Engineered Mouse (GEM) models utilizing known mutations identified in lung cancer patients (e.g. *KRAS*,* p53*,* ALK*) cause cancers to form orthotopically, however the long latency of cancer initiation and sporadic metastases^20–23^ reduce experimental feasibility. While conditional GEM models utilizing Cre-mediated recombination^24^ boost experimental feasibility by allowing for temporal and spatial control, the method of delivery is significant for evaluating progression and its overall clinical relevance. For instance, delivery of cre virus via nose or trachea initiate 10–200 adenomas/carcinomas^25^ possibly creating a drastically different TME from those seen in patients, impacting immune responses, and making it difficult to track disease progression from primary tumor to metastasis. Thus, the purpose of this paper is to report our methodology using direct orthotopic injection of a cell-specific Cre virus into the lung of a GEM, which induces a solitary tumor and spontaneously metastasizes, and can be adapted to different GEM/Cre virus combinations. To demonstrate our methodology’s experimental feasibility and clinical relevance, we present 2 models that uses a conditional GEM, Kras^G12D+^/p53^fl/fl^/myristoylated-p110α^fl/fl^-ROSA-gfp, developed by Dr. Steven Fiering^26^, using two specific lung Cre viruses, Ad5SPC which initiates cancer in alveolar Type 2 cells and Ad5CC10 which initiates cancer in the club cells, both developed by Dr. Anton Berns^27^. For both models, we provide data of how virus type and concentration affect successful tumor initiation and survival, serial uCT scans that distinguish primary tumors from metastasis, confirmed metastasis to specific organs at necroscopy, histology, and immune profiles comparing late vs. early stage cancer to signify importance of using tumor volume/stage when starting and evaluating anti-cancer drug response in mouse models (reflective of IASLC 5-year survival for cancer stages). In addition, we present our *evaluation* of how our models align with the IASLC tumor-node-metastasis (TNM) classification and histology criteria so that experiments using these models can readily and relevantly convey their expected clinical outcomes.

## Results

### Cancer initiation and intraoperative complications

**Fig. 1:**
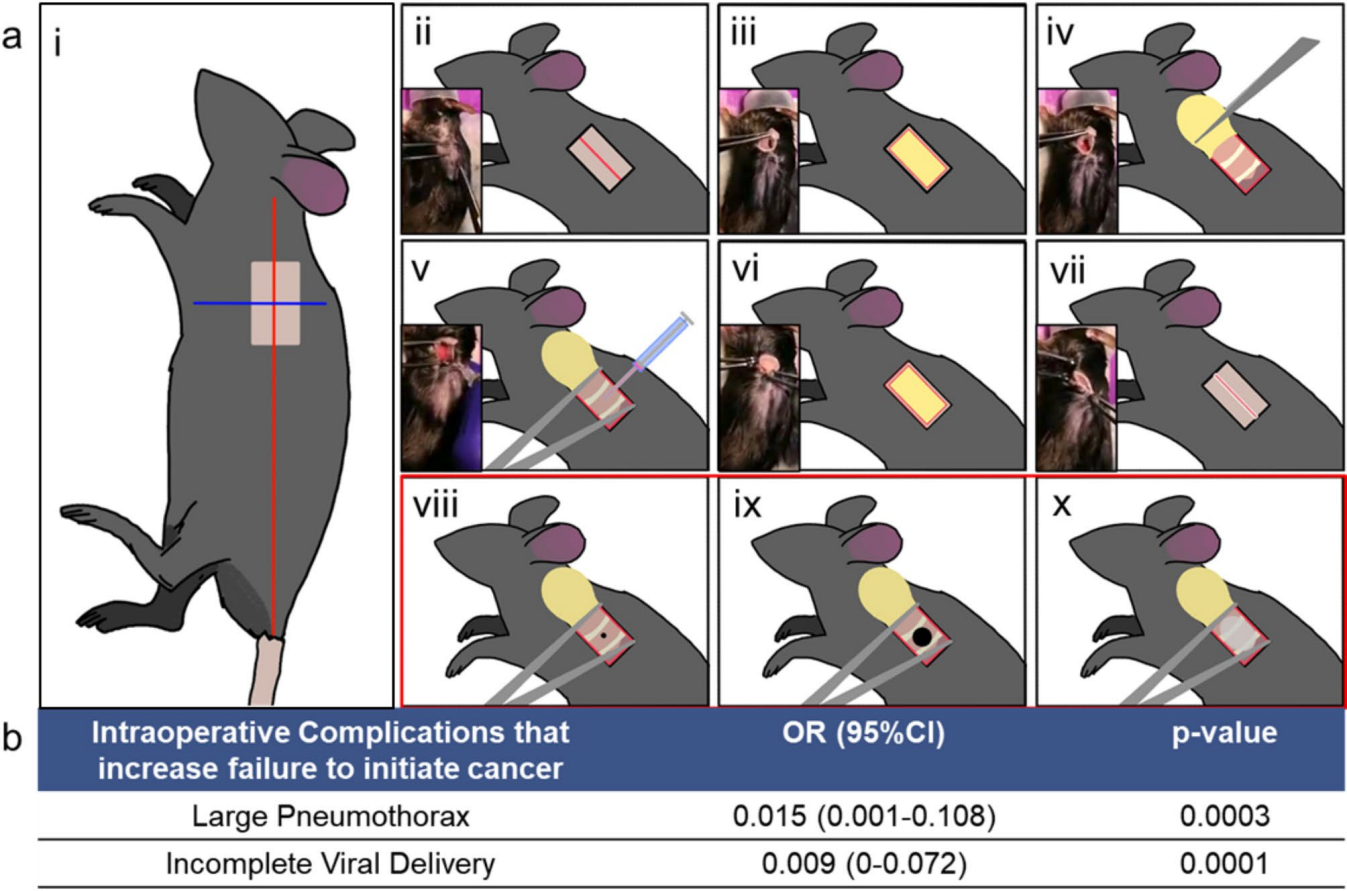
Surgical procedure of initiating a solitary lung tumor and Complications. (**a**) Step-by-step procedure for injecting Cre virus with direct visualization of the left lung. Details provided in Methods. (**b**) Reduced list of complications most likely to cause unsuccessful initiation of Lung Cancer. Full description of machine learning models used in Supplement Section S1.

We present two induced models of lung cancer that spontaneously metastasizes using the GEM, Kras^G12D+^; p53^fl/fl^; myristoylated p110α^fl/fl^ ROSA-gfp (abbreviated as KPP), developed by Sheen et al.^26^ The first model, S.KPP model was generated by injecting Ad5SPC Cre virus into the left lung initiating cancer in cells with Surfactant Protein C, specifically alveolar type 2 cells. Similarly, the second model, **C**.KPP model was generated by injecting Ad5CC10 Cre virus into the left lung initiating cancer in cells with **C**C10 protein, specifically club cells. Across all initiating cancer surgeries, post-operative survival rate was over 98%. Figure 1a demonstrates the steps of injecting Cre virus into the left lung (Details in Methods).

To specifically determine rate of success of initiating lung tumors, 10 mice (cohort 1) were injected with 10^6^ plaque forming units (pfu) of Ad5SPC cre virus (S**6**.KPP) (5M & 5 F) and 10 mice (cohort 2) with 10^5^ pfu of Ad5CC10 cre virus (C**5**.KPP) (5M & 5 F) at 3 months old, then uCT scanned 3 months post injection to confirm initiation. S6.KPP saw 8/10 (80%) and C5.KPP 9/10 (90%) successful lung tumor initiations, confirmed visually at necroscopy. When evaluating successful cancer initiation of S6.KPP and C5.KPP from other cohorts, 46/57 (81%) S6.KPP and 63/77 (82%) C5.KPP successfully initiated cancer.

During injection surgeries, 5 common intraoperative complications were recorded: bleeding, poor visualization, large pneumothorax, small pneumothorax, incomplete delivery. Analysis looking at each complication’s ability to predict failure to initiate cancer show that incomplete delivery or a large pneumothorax significantly reduced cancer initiation, with odds ratios being 0.015 (*p* = 0.0003) and 0.009 (*p* = 0.0001) respectively (Fig. 1b). See full description of analysis in Supplement Section S1 and Tables S1–S5.

### Histological analysis

#### Hematoxylin and Eosin (H&E)

**Fig. 2:**
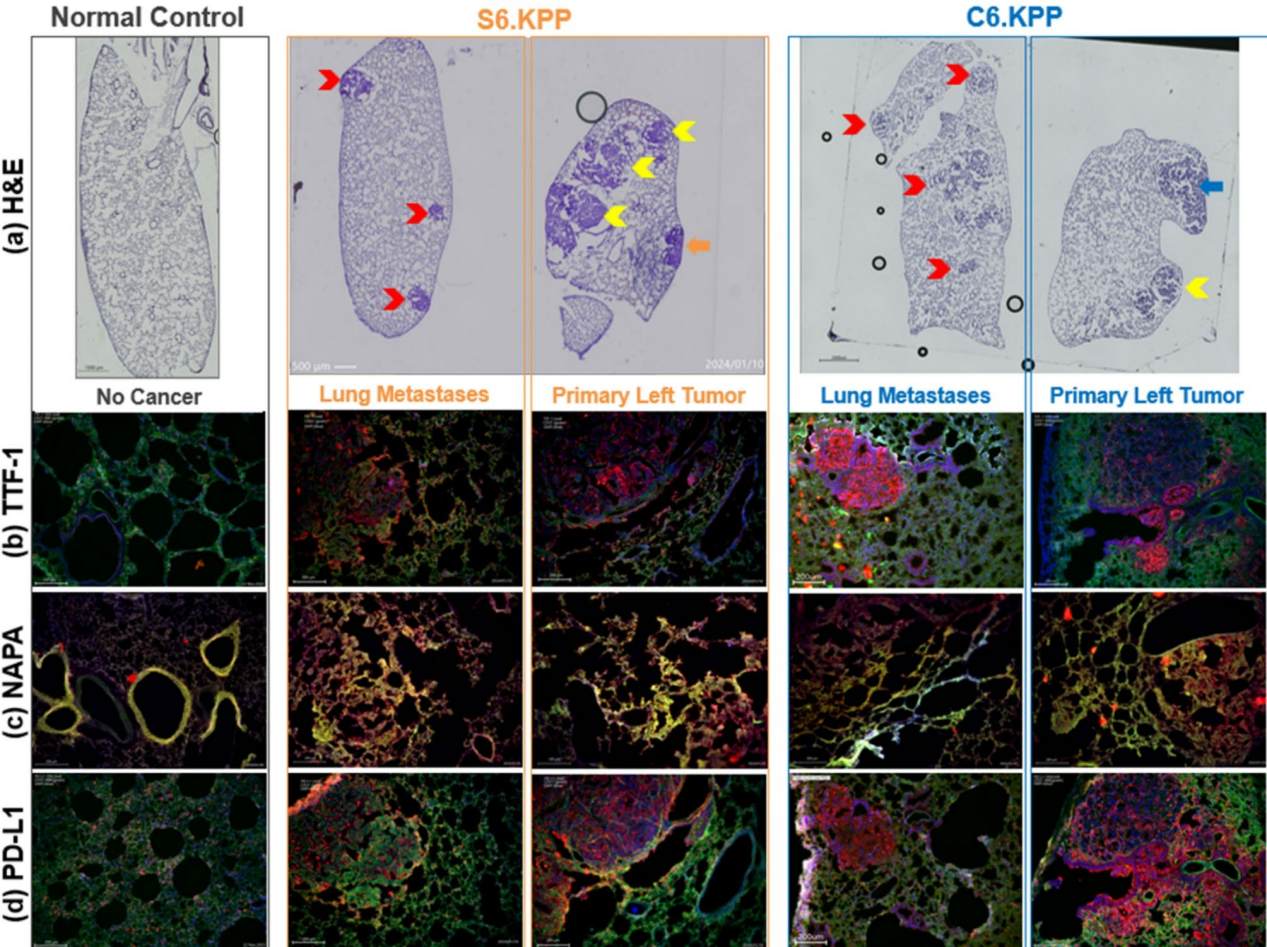
Histopathological analyses of Kras^G12D+^ p53^fl/fl^ myristoylated p110α^fl/fl^ ROSA-gfp injected with either Ad5SPC or Ad5CC10 Cre virus. Representative images of Normal Control (grey), S6.KPP (orange, primary tumor on right, lung metastases on left), C6.KPP (blue, primary tumor on right, lung metastases on left). (**a**) H&E staining reveals a lacy pattern in Normal Control lungs compared to solid primary tumors in S6.KPP (orange arrow) and C6.KPP (blue arrow), and ipsilateral (yellow chevron) and contralateral (red chevron) metastases. Images at 4X then stitched; scale bars = 1000 μm (Control, C6.KPP), 500 μm (S6.KPP). Both S6.KPP and C6.KPP primary tumors and metastatic tumors are strongly positive for (**b**) TTF-1, (**c**) Napsin A, and (**d**) PD-L1 compared to normal control. IF images: CD31 = green, DAPI = blue, protein of interest (TTF-1, Napsin A, PD-L1) = red; 10X; scale bars = 200 μm. For more information see Supplement Section S2. For 20x images, and single channel images, see Supplement Fig. S1.

In Fig. 2a and 4X (stitched), reveal significant differences between normal control lung and lungs from S6.KPP and C6.KPP mice. Primary left lung tumors in S6.KPP (orange arrow) and C6.KPP (blue arrow), ipsilateral left lung metastases (yellow chevrons), and contralateral right lung metastases (red chevrons), compared to normal lung control.

#### Immunohistochemistry

Histological analysis was selected based on what is performed clinically to diagnose lung adenocarcinomas, namely thyroid transcription factor 1 (TTF-1) and Napsin A aspartic peptidase (Napsin A)^28,29^. TTF-1 has a critical role in lung organogenesis^30^, and is normally expressed in type II pneumocytes, activating SPC^31^, and club cells, activating CC10^31^. However, given TTF-1’s high specificity for lung adenocarcinomas, positive TTF-1 staining in metastatic lesions strongly suggests a primary lung adenocarcinoma, as opposed to a potential unknown tumor metastasizing to the lung^32,33^. Napsin A has a role in processing surfactant protein B, and is normally found in type II alveolar cells^34^ and pulmonary macrophages, most likely due to phagocytosis^35^. However, given Napsin A is specifically overexpressed in lung adenocarcinoma and has minimal cross-reactivity with non-lung cancers, combined Napsin A/TTF-1 staining is standard clinical practice and useful to distinguish lung adenocarcinoma^36^.

Both S.KPP and C.KPP demonstrate heightened TTF-1 and Napsin A expression when compared to no cancer controls, specifically in/around primary and metastatic tumors. TTF-1 (red) colocalization with DAPI (blue) is consistent with its presence in the nucleus, appearing purple (Fig. 2b). Napsin A’s (red) stronger diffuse cytoplasmic staining in/around primary and metastatic tumors is also consistent with clinical lung adenocarcinoma, colocalizing more with CD31 (green) found on surface endothelial cells, appearing more yellow (Fig. 2c). Increased levels of both TTF-1 and Napsin A in both S6.KPP and C6.KPP suggests tumors produced in both models are Non-Small Cell lung cancer (NSCLC), specifically, lung adenocarcinoma.

PD-L1 is an immune checkpoint protein frequently upregulated in cancer cells to evade immune detection. The regions of dense cellularity that are positive for PD-L1 indicate that these tumors have high levels of PD-L1 expression, suggesting an aggressive or immune-evasive tumor phenotype. Both S6.KPP and C6.KPP show that PD-L1 (red) can colocalize with DAPI (blue, staining for nuclei) and the overlap (purple) confirms that PD-L1 is expressed in tumor cells, likely on the cell membrane or in the cytoplasm during transport and synthesis (Fig. 2d).

### Cre specific Cancer progression and metastasis

**Fig. 3:**
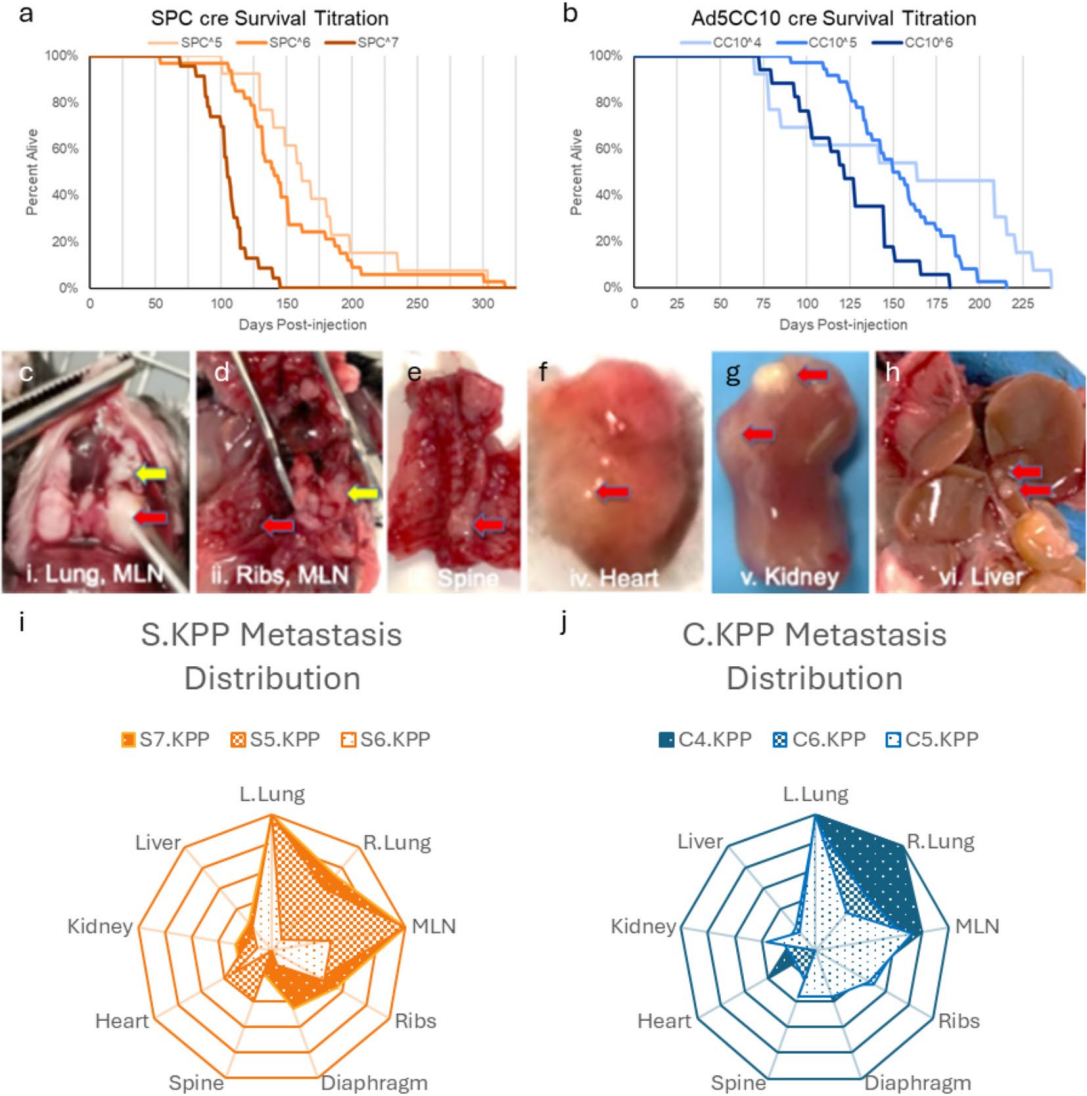
Survival and Metastasis Data by virus type and concentration. (**a**) S.KPP model. Survival analysis of mice injected with Ad5SPCcre at different concentrations (**b**) C.KPP model. Survival analyses of mice injected with Ad5CC10cre at different virus concentrations in the same GEM. Note that for a given concentration of virus, S5.KPP vs. C5.KPP there is no significant difference (t-statistic: -1.800, *p*-value: 0.072) in survival; however, with alpha being 0.05, power is 0.437. When comparing S6.KPP and C6.KPP there is a statistically significant difference (t-statistic: -2.762, *p*-value: 0.0059; with alpha being 0.05, power is 0.789) in survival. (**c**)–(**h**) Representative necroscopy images of C.KPP primary lung tumor and metastases to different organs. (**c**) Primary left lung tumor (i, red arrow) with visual confirmation of metastases to mediastinal lymph nodes (i and ii, yellow arrows) (**d**) metastases to ribs (ii, red arrow), (**e**) spine (iii, red arrow), (**f**) heart (iv, red arrow), (**g**) kidneys (v, red arrows), and (**h**) liver (vi, red arrows). (**i**–**j**) radar chart of metastasis site frequency with outermost ring representing 100% metastasis to the site by mice in that group, separated by virus type, (**i**) S.KPP and (**j**) C.KPP, and organized by concentration injected least to most metastasis.

Varying concentrations of both Ad5SPC and Ad5CC10 plaque forming units (pfu) cre virus resulted in different survival times and rates of metastasis. 69 mice were injected with Ad5SPC cre (cohort 3) at concentrations of 10^5^ pfu (S5.KPP *n* = 13), 10^6^ pfu (S6.KPP *n* = 33), 10^7^ pfu (S7.KPP *n* = 23) and 66 injected with Ad5CC10 cre (cohort 4) at concentrations of 10^4^ pfu (C4.KPP *n* = 13), 10^5^ pfu (C5.KPP *n* = 36), and 10^6^ pfu (C6.KPP *n* = 17), in approximate number of males and females (full description see Supplement Section S3). Ad5SPC cre virus concentration was inversely related to survival time in both mean (S5.KPP = 169d, S6.KPP = 149d, and S7.KPP = 102d) and median (S5.KPP = 160d, S6.KPP = 137d, and S7.KPP = 101d) (Fig. 3a). Ad5CC10 cre virus concentration was also inversely related to survival time in both mean (C4.KPP = 159d, C5.KPP = 145d, and C6.KPP = 118d) and median (C4.KPP = 155d, C5.KPP = 148d, and C6.KPP = 120d) (Fig. 3b).

The ability to metastasize is crucial for modern mouse models of cancer, especially since approximately 50% of patients with NSCLC are metastatic at diagnosis^37^. While other models have demonstrated an ability for cancer to spread to the lymph nodes, IASLC distinguishes cancer spread to the lymph nodes from metastasis using TNM guidelines. An additional cohort consisting of 59 mice (cohort 5), divided into S.KPP, 25 mice (12 F & 13 M) and C.KPP, 34 mice (18 F & 16 M) were injected at varying concentrations (Supplement Section S4). Metastasis was then confirmed at necroscopy (Fig. 3c–h), and the frequency of metastasis to common sites was recorded (Fig. 3i–j). As mice were always injected with Cre virus into their left lower lung, and it appears first on uCT scans, we were able to identify ipsilateral metastasis to upper left lung and contralateral metastasis to the right lung. Across all Ad5SPC and Ad5CC10 concentrations, ipsilateral metastasis occurred in nearly all mice (58/59, 98%), with the second most common site being the mediastinal lymph nodes (MLN) (44/59, 75%). Metastasis was less likely to ribs (28/59, 47%), diaphragm (17/59, 29%), heart (12/59, 22%), kidneys (24/59, 24%), spine (12/59, 20%), and liver (12/59, 20%). Interestingly, contralateral lung metastasis was more likely at the low and high concentrations of both Ad5SPC and Ad5CC10 cre viruses than the intermediate concentrations (Fig. 3i–j).

### Cancer progression mirrors clinical classification guidelines

The IASLC 8th edition of the TNM classification system for non-small cell lung cancer begins with primary tumor ranked as T0 - T4, with distinctions being made on size and thoracic invasion. Regional lymph nodes are ranked as N0 - N3, from no regional node metastasis to ipsilateral pulmonary, ipsilateral mediastinal, and contralateral mediastinal. Distant metastasis, ranked as M0 - M1c, are classified by metastases to either the contralateral lobe or extra thoracic organs. While previous orthotopic implantation models were able to report invasion to regional lymph nodes at necroscopy^38^, a S6.KPP cohort of *n* = 12 (cohort 6), gender and age matched (6 M, 6 F, 3 months at injection), was able to demonstrate stages laid out by the TNM classification system. See further details in Supplement Section S5.

**Fig. 4:**
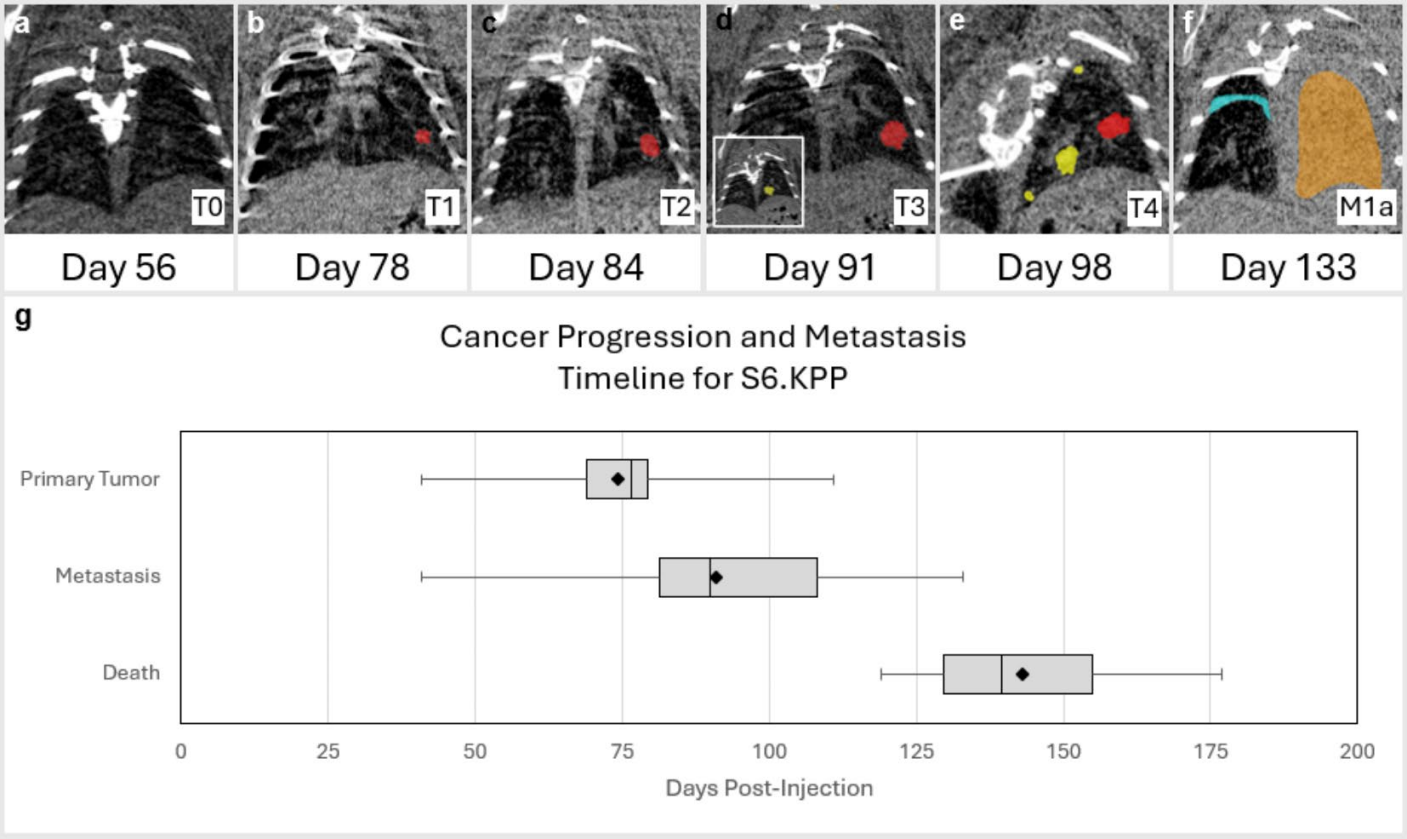
Cancer Initiation, Progression, and Metastasis for S6.KPP Mouse Model. (**a**–**f**) Representative uCT scans chronologically showing the cancer progression post-injection from (**a**) no tumor, (**b**) primary tumor initiation (red), (**c**) growth in size before, (**d**) ipsilateral metastasis (yellow) (considered to be same lobe), (**e**) ipsilateral metastasis (yellow) (different lobe), to (**f**) metastasis as defined under IASLC TNM guidelines (cyan) (malignant pleural effusion in contralateral lung). (**g**) Timeline depicting the typical progression of the S6.KPP model. The plot shows the time to primary tumor detection, appearance of either ipsilateral or contralateral metastasis, and death post-injection. Indicated are median, interquartile range, minimum and maximum days for each event, with the diamond representing the mean.

Longitudinal weekly uCT scans post-injection (Fig. 4a–f) shows the lung with no evidence of tumor (T_o_), before the first tumor appears (T_1_), determined as the primary tumor. The primary tumor progresses in size (T_2_-T_4_), ipsilaterally metastasizes(T_4_), eventually obscuring the entire lung and causing malignant effusion in the contralateral lung (M_1a_). While T_3_ and T_4_ are differentiated by size, they also differ in placement of tumor nodules, namely the same or separate lobe of the ipsilateral lung respectively. While this distinction is impossible to make in mice with only 1 left lung lobe, T_4_ was stated by the appearance of the third tumor present in the ipsilateral lung. Identifying tumor nodules in the lymph nodes was difficult to confirm in uCT scans but were confirmed at necroscopy. Of this S6.KPP cohort, the primary tumor was distinctly present (mean = 74.3d, median = 76.5) before the first metastasis was documented (mean = 91d, median = 90d), and had similar survival time (mean = 143d, median = 139.4) post injection to expected time established by the survival curves of the same virus and concentration (Fig. 4g).

### Immune profiles of mice and men display similar lung cancer behavior

To further evaluate similarities of our orthotopic injection models to clinical patients, flow cytometry was conducted on a cohort of 76 mice (cohort 7) of frequently observed metastatic sites, divided into 3 groups: S7.KPP-Early Stage (*n* = 19,10 F & 9 M, tumor volume 3–8 mm^3^), S7.KPP-Late Stage (*n* = 17, 15 F & 2 M, tumor volume 50–85 mm^3^), and No Cancer (*n* = 40, 20 F & 20 M).

**Fig. 5:**
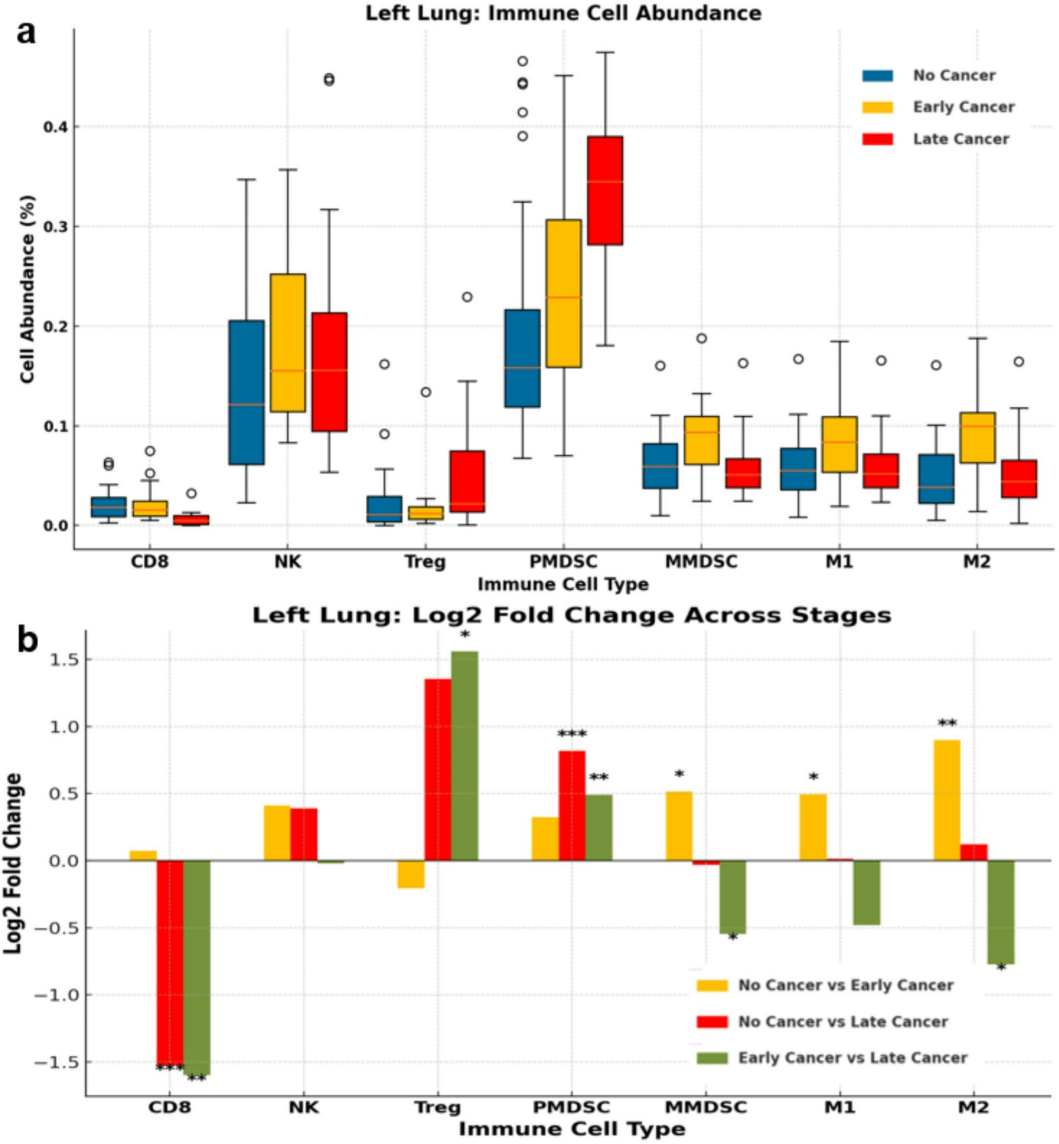
Immune Profiles of Left Lung comparing S7.KPP-Late vs. S7.KPP-Early vs. No cancer. (**a**) percent of cell abundance for CD8 (CD45+, CD3+, CD8+); NK (CD45+, CD3-, CD314+, CD49b+); Treg (CD4+, CD25+, FoxP3+); PMDSC (CD11b+, Ly6G-Hi, Ly6C-Lo); MMDSC (CD11b+, Ly6G-Lo, Ly6C-Hi); TAM/M1 (CD11b+, F4/80+, MHCII-Hi); TAM/M2 (CD11b+, F4/80+, CD206+) via flow cytometry, in three groups: no cancer in blue, early-stage cancer (3–8 mm^3^) in yellow, and late-stage cancer (50–85 mm^3^) in red. (**b**) Comparison of three groups using Log2 fold change. Significant T-Test *p*-values shown; *p*-value *≤* 0.05 = (*), *p*-value *≤* 0.005 = (**), *p*-value < 0.001 = (***).

When comparing the ipsilateral left lung of each group to one another, statistically significant differences in immune cell populations emerged, similar to clinical observations. For instance, late-stage cancer mice had significantly higher levels of regulatory T cells (Tregs) (Fig. 5), which was also observed in patients with advanced stages of NSCLC, and has been associated with tumor growth and metastatic potential^39,40^. Late-stage cancer mice also had increased levels of polymorphonuclear myeloid-derived suppressor cells (P-MDSCs) (Fig. 5) when compared to early-stage or no cancer mice, which was also observed in patients with metastatic disease^41,42^.

Late-stage cancer mice demonstrated decreased populations of CD8 + T cells and M1 macrophages when compared to early-stage or no cancer mice (Fig. 5). This is akin to clinical observations where M1 macrophages, CD8 + T cells, and various subsets tends to decrease as NSCLC advances to later stages^43^ especially when compared to early-stage patients^44,45^.

While monocytic myeloid-derived suppressor cell (M-MDSC) and M2 macrophage populations tend to increase with disease progression in humans^46,47^, exact parallels were not apparent in our murine immune profiles, suggesting that different mechanisms of immune suppression may be predominant in the ipsilateral left lung. However, early-stage accumulation of M-MDSCs was associated with elevated concentrations of inflammatory mediators involved in MDSC migration to and activation in the tumor microenvironment^41^. While this migration continues in more advanced stages, such mobilization relies upon tumor-derived factors to accumulate in the TME where they inhibit T-cell proliferation and activation, often associated with decreased levels of CD8 + T cells^48^. M2 macrophage populations are similarly tied to the tumor’s ability to secrete various cytokines and growth factors which recruit and polarize macrophages towards the M2 phenotype^49^. This suggests that while the tumors produced initially recruit M-MDSCs and M2 macrophages in early stages, mechanisms within our model to utilize them as primary immunosuppression may not be present or needed in later stages, at least in the ipsilateral lung. Other metastatic organ immune profiles are available in Supplement Section S6.

### Rate of Tumor Progression to Cisplatin and Immunotherapy May depend on initial size

Anti-cancer drugs have and continue to be the predominate non-curative life extending treatment offered to patients with advanced stage lung cancer. This includes chemotherapy such as cisplatin, estimated to be used in ~ 60–70% patients with lung adenocarcinoma^50,51^, and immunotherapy, estimated to be used ~ 31% of lung cancer patients are treated with immunotherapy^52^ (often as first line in combination with chemotherapy). As newer classes of targeted therapies are developed, such as *KRAS*, *P53*, *PI3K* and *EGFR* inhibitors^53^, and more anti-lung cancer drugs are developed to specific genetic targets, it is important to develop immunocompetent mouse models capable of responding to treatments.

**Fig. 6:**
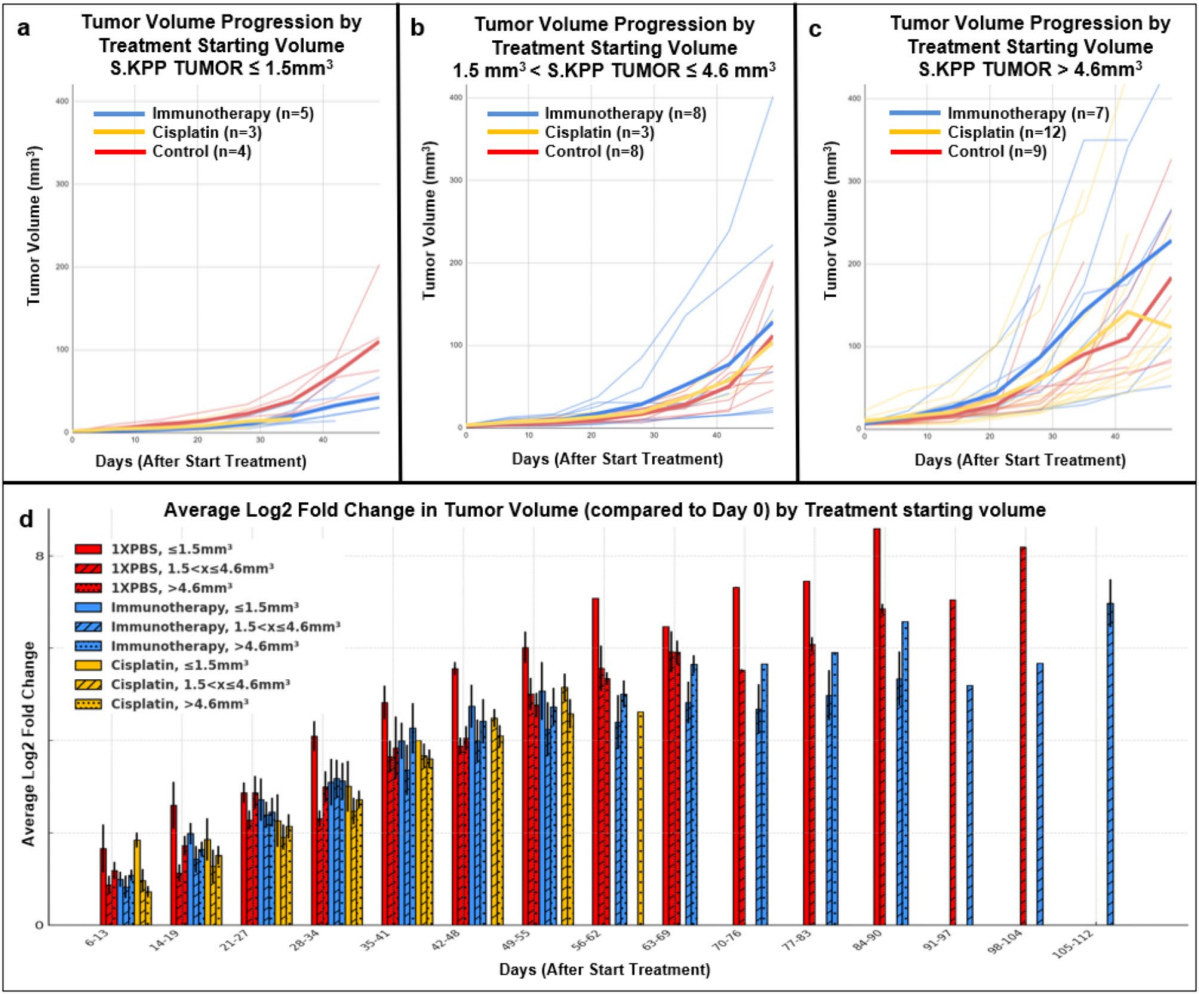
Rate of tumor volume progression of S.KPP mice treated with immunotherapy (alternating anti-PD-1 and anti-PD-L1) vs. cisplatin vs. control. (**a**–**c**) Each treatment mouse is plotted only once (thin red lines are individual mouse rate of tumor progression treated with control; thin blue lines are individual mouse rate of tumor progression treated with immunotherapy; thin yellow lines are individual mouse rate of tumor progression treated with cisplatin). Control mice could be used again in a larger starting size group if it showed a uCT scan within starting size parameters to increase statistical significance. (**d**) Average log2 fold change in tumor volume (compared to starting volume on Day 0, when treatment started) is plotted by treatment type (1XPBS, Immunotherapy, Cisplatin) over time to visualize whether treatment might show an existing difference in small (≤ 1.5 mm^3^—solid bars), medium (1.5 < x ≤ 4.6 mm^3^—hash bars) and large (> 4.6mm^3^—dotted bars). For information on mice included see Supplement Table S7.

To assess if our models produce tumors that are responsive to anti-cancer drugs and whether initial tumor size when starting treatment was significant, tumor volumes were analyzed over a 7-week treatment course, in which treatments were started at different initial tumor sizes (Cohort 8). Treatment groups were Immunotherapy: 20 mice (9 F & 11 M), Cisplatin: 18 mice (9 F & 9 M), and Control (no treatment): 9 mice (4 F & 5 M) (Fig. 6). Mice were separated into 3 starting volumes; small (starting tumor volume, x *≤* 1.5mm^3^) (Fig. 6a), medium (starting tumor volume, 1.5mm^3^ < x *≤* 4.6mm^3^) (Fig. 6b), and large (starting tumor volume, x > 4.6mm^3^) (Fig. 6c). We alternated anti-PD-1 and anti-PD-L1 therapies to mitigate adverse effects and enhance therapeutic outcomes. While it is clinically standard to combine cisplatin therapy with etoposide or pemetrexed, we are currently acquiring foundational insights of monotherapy before embarking on combination therapy studies that may result in significant adverse effects that are not easily assessed in mice. For additional details about treatment regimen see methods.

When plotting tumor progression, due to limited number of mice, S6.KPP and S7.KPP were combined as doubling time of the Control tumor volumes were similar in the 7-week period analyzed, and some control mice were included in multiple starting volume groups as they would never receive treatment; additional details in Supplement Section S7. Power analysis showed low statistical power, so definitive conclusions cannot be reported, but patterns were observed. The *S.KPP model* showed primary responses to both immunotherapy and cisplatin when started in small tumors, indicating that tumors produced by this model are responsive to treatments and are thus useful for testing anticancer therapies. Treatments started at medium or large tumor volumes had no effect or increased tumor progression, indicating initial tumor size does affect tumor response to treatment.

## Discussion

Herein we report on conditional mouse models of lung adenocarcinoma, using direct orthotopic injection of Ad5Cre virus into our GEM. Injection of two different viruses (Ad5SPCcre and Ad5CC10cre) demonstrate consistent tumor initiation rates regardless of virus type, with negligible post-operative complications. Resultant tumors and metastasis were validated using clinical markers of lung adenocarcinoma; TTF-1 and Napsin A and are PD-L1 positive. uCT scan evidence demonstrates primary tumors progress similarly through IASLC stages from solitary tumor to spontaneous thoracic metastasis, with extrathoracic metastasis confirmed at necroscopy. Using flow cytometry, immune profiles of cancer induced mice generally reflected clinical observations at early and late stages, and resultant tumors were also preliminarily responsive to treatments when controlling for initial size when treatment was started.

Models derived from this methodology draw important parallels to clinical observations previously overlooked by murine lung cancer models, specifically its ability to metastasize and metastasis location. Preceding models of metastatic lung cancer in mice, orthotopic or otherwise, share common issues with being unable to temporally distinguish primary tumors from metastasis. While previous models of lung cancer via orthotopic injection report tumorigenesis, metastatic spread was limited to the lymph nodes^38^, which clinically is maintained separate from metastasis. In this study, the use of specific cre viruses, combined with the p110α deletion, metastasis occurs 100%. However, further investigation is needed to elucidate the significance of p110α deletion compared to other mutations, as well as mechanisms of how it influences metastases. In this study, the use of specific cre viruses, combined with the p110α deletion, metastasis occurs 100%. However, further investigation is needed to elucidate the significance of p110α deletion compared to other mutations, as well as mechanisms of how it influences metastases. Furthermore, by utilizing uCT scans, clear progression of IASLC stages was demonstrated making possible for the first time, the opportunity to infer clinically paralleled results in preclinical trials.

Additionally, our model demonstrated similar immune profiles of tested immune cell populations in early and advanced stage cancer patients, suggesting recapitulation of similar mechanisms of immunosuppression or evasion observed in humans. This has significant implications for treatment discovery as accurate recapitulation of disease initiation and progression can greatly focus and test new treatments. Additionally, preliminary results indicate tumors are responsive to cisplatin and immunotherapy, with starting tumor volume having a significant effect on treatment response. This lends support to what is done clinically, which is instituting treatment based on stage, which factors size, not time, which is frequently done in preclinical models.

Limitations of our methodology are the expertise and cost of initiating tumors via orthotopic injection, as well as monitoring orthotopic lung tumors. However, by detailing our surgical procedure to orthotopically induce tumors, and deliberate protocol choices, such as not to intubate the mouse or use stereotactic equipment to keep costs low, we enhance experimental feasibility and allow for high throughput given high success rate of initiation. In addition, by demonstrating how different virus types and concentrations produce different survival times, metastasis rates, and metastasis distribution, this may facilitate adoption of this new method and allow investigators greater flexibility of inducing tumors based on primary tumor progression rate, metastasis, or survival time, in an immunocompetent mouse, with greater clinical relevance. While we did not trace or quantify the initially transformed cells to evaluate the specificity and targeting efficiency of the Cre viruses, establishing this methodology as state of the art now, would be significant given the growing availability and reliability of GEMs and Cre viruses, facilitating diverse and novel investigations, especially as more specific targeted therapies are being developed. While tracking thoracic disease progression can be challenging and costly via uCT scans, and more so for extrathoracic metastasis, the conditional initiation of tumor demonstrated remarkable consistency in timing of primary tumors and metastasis. This allows investigators to greatly reduce the window in which uCT scans are required after establishing baselines. We anticipate this methodology to be further improved to facilitate greater translation of basic science to clinical trials by fundamentally shifting how lung cancer is studied.

We intend to further advance this methodology by assessing heterogeneity of tumors as it progresses, developing protocols for surgical debulking, chemoradiotherapy, and high throughput/cost effective methods to identify distal extrathoracic metastases. Lastly, we are developing a classification system within mice that aligns with the IASLC/WHO TNM lung cancer staging to predict survival and incorporate treatment effects, enhancing clinical relevance.

## Methods

### Ethical considerations and mouse monitoring

The study protocol was reviewed and approved by the institutional animal care and use committee (IACUC) at The University of Pennsylvania and at The Miami VA Healthcare System. All experiments were performed in accordance with relevant guidelines and regulations at each institution and with ARRIVE guidelines. Humane endpoints were established in accordance with standard operating procedures set by the institutional IACUC, including daily observations of respiratory distress, hunched back, decreased movement, paralysis from spinal metastasis, weight loss threshold of > 10% body weight and poor grooming. Any euthanasia was carried out via CO_2_ chambers, and secondarily confirmed via cervical dislocation.

### Genetically engineered mice (GEM)

Our conditional GEM, Kras^G12D+/−^/p53^fl/fl^/myristoylated-p110α^fl/fl^-ROSA-gfp, was developed by Sheen et al.^26^ (mice were gifted by Jose R. Conejo-Garcia). In brief, Kras^tm4Tyj^ and Trp53^tm1Brn^ mice were intercrossed with a C57BL/6 Cre-inducible myristoylated-p110α-ROSA-gfp mice^24,54^. By back-crossing to C57BL/6, mice are immunocompetent.

### Mouse husbandry

For breeding, one male, triple transgenic Kras^G12D+/−^ mouse was housed with two female, triple transgenic Kras^G12D−/−^ mice. After babies were born, sunflower seeds were given to promote lactation. When cages became overcrowded, but babies were not weaned, mice were moved to larger hamster-sized cages to provide adequate space for larger litter. Hamster-sized cages were maintained with identical bedding, food, and water sources. Mice were weaned at 21–24 days old, ear tagged, and tail clipped for genotyping at 6–8 weeks old using PCR. All mice in Cohorts 1–9, were homozygous for p53^fl/fl^ deletion and myristoylated p110α^fl/fl^ ROSA-gfp. Mice were housed by gender, age, and genotype into cages of 5; age range within 1 cage < 1 week apart. Living conditions were strictly monitored and remained consistent throughout the experimental period. They were provided a diet of water and rodent diet pellets. Cages were changed weekly by vivarium staff, and the mice were visually inspected daily. Any mice displaying signs of distress, such as improper grooming or severe weight loss (> 15% total body weight) were promptly harvested and/or euthanized based on their experimental group assignments.

### Viral vector production and preparation

Ad5mSPC (Iowa Vector Core, cat no. VVC-Berns-1168)^27^ and Ad5CC10 (Iowa Vector Core, cat no. VVC-Berns-1166)^27^ Cre viruses were aliquoted and stored at -80 °C. On the day of injection, viruses were activated according to manufacturing guidelines. In brief, the virus is diluted in DMEM adjusting the volume of DMEM depending on the intended injection concentration and volume. Add 0.25 M CaCl2 such that the activated virus has a final concentration of 20mM CaCl2, mix, and allow to sit for at least 20 min before use. The volume of virus and DMEM will depend on the titer provided by the manufacturing facility. Timing of each dose of Cre virus is recorded for each mouse.

### Survival surgery of intrathoracic injection of cre virus

#### Step 1: surgical field preparation

Person #1 deeply anesthetizes the mouse using continuously inhaled isoflurane mixed with O2 at a flow rate of 1.5 L/min. When pedal reflexes are absent, a subcutaneous (SQ) injection of 0.05 mg/kg buprenorphine and 5 mg/kg meloxicam in the fat pad between the ears is administered and hind toenails are trimmed to prevent the mouse from scratching and reopening their incision post-operatively. The mouse is then positioned with their right side down such that the left lung is up. We specifically inject the left lung because it has one lobe, and it is easier to identify metastases. Landmarks for the surgical site is visualized: a longitudinal axis is drawn from the base of the left ear to the tail, and a latitudinal axis is perpendicular to the longitudinal axis below the left arm, such that the intersection coincides with the largest amplitude of breathing movement. The mouse is shaved at the intersection, ~1 cm wide and ~ 2 cm long using a 0.5 mm shaver, after which the surgical site is sprayed with 70% isopropyl alcohol alternated with chlorhexidine and repeated 3 times. When ready to begin surgery, adjust the isoflurane mixer to keep respiratory rate as low as possible.

Person #2 is keeping surgical notes of Mouse ID including time of injection (relative to Cre virus preparation), complications of injection, getting the next mouse ready, monitoring previous mice post-operatively, drawing up the Cre virus. *Cre virus* Gently pipette the Cre virus solution up and down 5 times, then pulling up 10uL of Cre viral solution onto the lid of the Eppendorf tube. Using an insulin syringe that has a 29G 1/2” needle, draw up the 10uL of Cre virus solution, being very careful to not introduce air within the syringe as it will cause a large pneumothorax and/or incomplete viral delivery that significantly impacts cancer initiation; the insulin syringe is placed on the table near Person #1.

#### Step 2: incision and intrathoracic injection of cre virus

Person #1 uses Adson dressing serrated forceps to make a small fold in the skin (Fig. 1a, i) and iris scissors are used to make an incision ~ 0.5 cm (folded) so it is ~ 1 cm unfolded. Blunt dissection with scissors is conducted to visualize the fat pad. Adson serrated far tooth forceps are used to pick up the fat pad (Fig. 1a, ii) and scissors release the fat pad along the bottom and sides until able to hold the flap of fat (Fig. 1a, iii). Once the lung is visualized (Fig. 1a, iv) hold the incision open with curved forceps, and identify site of injection: midaxillary line, ~ 2 ribs above the bottom edge of the lung, in between 2 ribs.

Person #1 will pick up the syringe and hold the needle tip at a 90° angle, perpendicular to the lung with the bevel up; carefully count 1–2 breaths of the mouse, and immediately after a breath inject the needle into the lung (Fig. 1a, v), ~1 mm more than the length of the entire bevel. If the bevel if not entirely within the lung, this will result in an incomplete delivery of virus. With the needle in the lung, after allowing the mouse to take 1–2 breaths, immediately after a breath, Person #2 will slowly push the plunger of the insulin syringe. After Person #2 releases the plunger, Person #1 will hold the needle in the lung for an additional ~ 3–4 breaths so fluid does not escape, then retract the needle out at the same angle it was injected.

#### Step 3: closing the incision

Person #1 uses forceps to gently pull the fat pad down and cover the injection site (Fig. 1a, vi), being careful there are no stray hairs near the incision site. Using curved forceps in left hand and Adson dressing serrated forceps in right hand, approximate the skin edges of the incision so they touch (Fig. 1a, vii) and pull the length of the incision site in opposite directions (left hand toward ear and right hand toward tail). Once approximated, open the incision slightly and drop surgical glue across the entire length of the incision site (Fig. 1a, viii). Using silicon tips (we use the needle cap from the insulin syringe), gently approximate the incision site without touching the glue (Fig. 1a, ix). The glue is usually cured within 8 s, but we found that the incision site can still open. At this point, turn off the anesthesia, then manually approximate the incision closed using a gloved hand lightly sprayed with 70% isopropyl alcohol to prevent the glove from sticking to the surgical site and opening the wound. We then add a second layer of glue on top of the incision and again manually approximate the incision using a gloved hand sprayed with 70% isopropyl alcohol; this is repeated with a third layer of glue. The final result is a closed incision site (Fig. 1a, x).

#### Step 4: postoperative recovery

Person #2 puts the mouse into a clean cage with the incision facing up, but the mouse is not supine. A red heat lamp that keeps ~ 2/3 of the cage warm and ~  1/2 of the cage cool (not under a red heat lamp); this allows the mouse to be kept warm while coming out of anesthesia but has the liberty of move to a cooler location if it is too warm. With careful monitoring, the mouse will start to move within minutes and fully move within 5 min. We keep all mice from one cage to recover together and Person #2 closely observes the mice until they all resume normal activity, including eating and drinking. For the next couple of days, incisions are inspected; if an incision is open, the mouse is placed under anesthesia and the wound is inspected. If the wound is superficial, the incision is cleaned and reapproximated with surgical glue, given another dose of buprenorphine 0.5 mg/kg XR with meloxicam 5 mg/kg and separated from cage mates until the wound heals. If the wound is deep or has an abscess, the mouse is sacrificed.

Preventing wound dehiscence is a major non-lethal complication that can cause infection or a chronic wound that does not heal, which not only alters the TME and immune response, but in some instances require euthanasia. Initially, we had significant wound dehiscence but this was largely solved by (1) cutting the toenails under anesthesia so when the mouse scratched a healing wound, it would not reopen; (2) closing the wound with surgical glue for a total of 3 times; (3) removing hard-plastic enrichment devices in home cages for about one week to prevent sharp edges catching the acute wound and opening it.

We initially used a Hamilton syringe with the shortest bevel (a blunt edge will not get through the pleural linings) but found that the needle easily dulled after repeated use and contributed to a larger pneumothorax. We eventually chose to use insulin syringes because they not only have no hub (therefore no dead space), but also, they have a high gauge needle that leaves a very small pneumothorax and are widely available and inexpensive that keeps costs down and increases feasibility. Furthermore, to adapt our method to other GEM + Cre virus combinations, we recommend doing a full titration of virus concentration to optimize timing of metastases, survival and response to treatment.

### Serial micro computed tomography (µCT) to quantitate 3D Tumor volumes

Most mice underwent micro computed tomography (µCT) of the chest one month following survival surgery and typically every 1–2 weeks thereafter (there were times when mice could not be scanned due to issues out of our control). At Institution A, we utilized the Molecubes X-cube with the following settings: 50 kV, 440 uA, 480 exposures with 125 ms/exposure for a total of 48mGy per scan, reconstructed to 100 μm. At Institution B we used the Bruker Skyscan 1172 scanner (Bruker Corp., Billerica, MA) with the following settings: 50 kV, 400 uA, 180 exposures with 70 ms/exposure for a total of 28 mGy per scan, reconstructed to 35 μm.

For image acquisition, mice were anesthetized using continuously inhaled isoflurane at 1.5 L/min administered via nosecone. Within the scanner, mice were placed prone with a respiratory sensor pad under the abdomen and physiologic monitoring software employed for respiratory gating. Active scan time per mouse was largely under three minutes, radiation was localized to the chest only, allowing for more views with greater resolution and lower radiation. Images acquired from both scanners were analyzed using ITK snap^55^, a freely available software www.itksnap.org to view and segment tumor volumes in the axial, sagittal, and coronal planes. Manual segmentation of the largest diameter in each plane was used to calculate an oval volume.

#### Treatment: immunotherapy and cisplatin

After tumor volumes were calculated mice received either weekly intraperitoneal injections of immunotherapy or cisplatin. Immunotherapy Mice were injected with anti-PD-1 (BioXCell, InVivoMAb anti-mouse PD-1 (CD279), #BE0033-2) on Tuesdays, and anti-PD-L1 (BioXCell, InVivoMab anti-mouse PD-L1 (B7-H1), #BE0101) on Thursdays; both at a dose of 10 mg/kg. This schedule came about from initially treating mice with monotherapy (anti-PD-L1 or anti-PD1) which proved to be ineffective in slowing tumor progression, or simultaneous combination therapy (anti-PD-L1 and anti-PD1 on the same day) which resulted in adverse events in some of the mice. This led us to adopt an alternating schedule of immunotherapy which has demonstrated no adverse events and slowed tumor progression and increased survival. Cisplatin Mice were injected with cisplatin (medical grade, provided by pharmacy at Institution B) on Tuesdays, at 4 mg/kg. Control Mice were injected with sterile PBS, also given on Tuesdays and Thursdays.

#### Necroscopy, immunofluorescence, immune profile

Necroscopy was performed on deeply anesthetized mice with no pedal reflexes using isoflurane. After lung tissue was cryopreserved, to preserve lung architecture during immunohistochemistry protocol, adherent cryofilm (Section-lab, Hiroshima, Japan)^56^ was applied directly to the tissue then sliced. To obtain immune profile, we collected six organs from each mouse and stained six immune cell populations. Details in the Supplement.

## Electronic supplementary material

Below is the link to the electronic supplementary material.

Supplementary Information.

## Data Availability

All other data supporting the findings of this study is provided within supplementary files or are available from the corresponding author on reasonable request.
