# Supplementary material for: Methodology of murine lung cancer mimics clinical lung adenocarcinoma progression and metastasis
Source: Sci Rep. 2025 Feb 28;15:7127. doi: 10.1038/s41598-025-90344-1 (PMC11871348; doi:10.1038/s41598-025-90344-1)

**Supplement**

**Section S1. Results: Experimental feasibility: intraoperative complications of initiating orthotopic lung cancer**

***Cohort 1 and 2: intraoperative complications*** Mice were combined for Cohort 1 and 2 to analyze the impact of intraoperative complications on failure to initiate a primary lung tumor, using a two-step process.

**Step 1:** We categorized all mice to have one or more of the following five different intraoperative complications:

1. *Bleeding*: larger bleed occurred if a small vessel was cut when releasing the fat pad to visualize lung, this clots within seconds of pressure; smaller pinpoint blood drop occurred when pulling needle out of the lung.
2. *Poor visualization*: defined as suboptimal visualization of needle in the lung either due to hand position, lighting or mouse respiration.
3. *Large pneumothorax*: defined as drop in lung when pulling out needle out of the lung; we hypothesize this may be due to air in the needle or possibly inadvertent entry into a large airway.
4. *Small pneumothorax*: defined as an expected small hole in lung where needle entered the lung.
5. *Incomplete delivery*: defined as part of the viral load is outside of the lung; we hypothesize this may be due to sedation not being deep enough or needle bevel is partially in and partially out of lung.

Of the injected, one mouse did not survive beyond twenty-four hours of the procedure due to the development of postoperative seizures and was excluded from the final analysis (seizure etiology remains unexplained).

**Step 2:** We used advanced statistical analyses to determine which intraoperative complications were most likely to result in failure to initiate cancer. An overview of this analysis is as follows:

1. *Descriptive statistics:* Baseline characteristics (**Table S1)**
2. *Determine intraoperative complications that impact cancer initiation*: We then used 3 different models to determine which intraoperative complications most likely contributed to the failure of initiating primary lung cancer:

- **Model 1** Full Model using logistic regression to assess individual and interaction effects of complications (**Table S2**)
- **Model 2** Reduced Model used stepwise logistic regression to identify significant complications (**Table S3**)
- **Model 3** Classification Models using four different machine learning models to validate findings (**Table S4**, **S5**).

**Descriptive statistics (Table S1)** We provide a distribution of intraoperative complications divided by failure or success of initiating lung cancer. A univariate analysis to investigate the effect of each of the five intraoperative complications, including mouse gender; both large pneumothorax and incomplete viral delivery were significant.


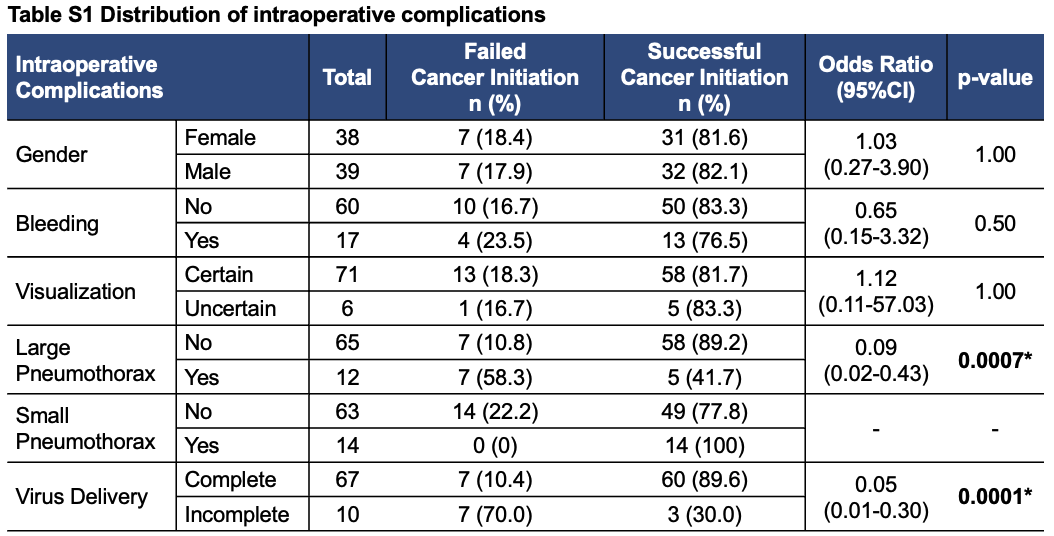


**Model 1** (**Table S2)** We developed a full model using logistic regression that provides a coefficient of an individual intraoperative complication or interaction of 2 intraoperative complications. The coefficient provides the relative contribution to failure of initiating primary lung cancer denoted as a negative sign, or success to initiating primary lung cancer denoted as a positive sign. We used the following equation:


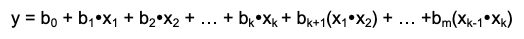


Large pneumothorax had a coefficient of -2.67 with a small 95% confidence interval resulting in a significant p value of 0.0453. Incomplete viral delivery had a coefficient of -4.06, also with a small 95% confidence interval resulting in a significant p value of 0.0010.


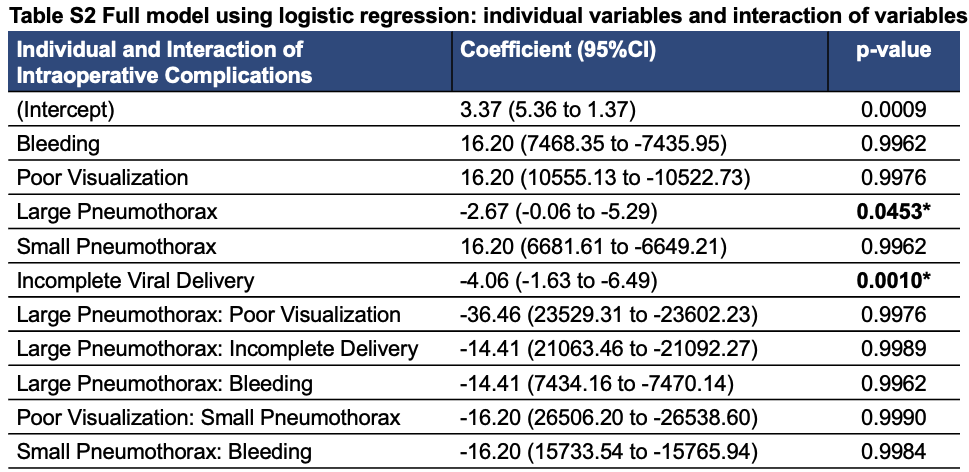


**Model 2 (Table S3)** After establishing a full model to investigate the main and interaction effects, of individual intraoperative complications and interactions, we then conducted a bi-directional stepwise regression utilizing Bayesian Information Criterion (BIC) as the selection criterion to develop a reduced model (Model 2). In short, all individual intraoperative complications and every possible combination of two intraoperative complications were run through the model and ranked in order from most significant to least significant contribution to failure of initiating primary lung cancer. We then deleted the least significant individual/combination intraoperative complication, then reran the model. This was repeated until the final two intraoperative complications remained; this is the true coefficient. Again, both large pneumothorax and incomplete viral delivery were significant.


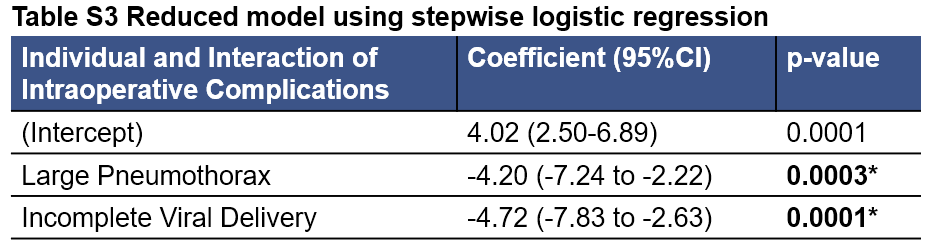


**Model 3 (Table S4)** We performed a cross validation using an unbiased approach that employed 4 different models to determine the importance of each intraoperative complication to classify failure of cancer initiation. The receiver operating characteristic curve (ROC) for logistic regression, neural network, K-nearest neighbor and classification/regression tree models are presented that evaluate the variable importance of each intraoperative complication on failure to initiate lung cancer; variable importance was converted to a 0-100 scale (the higher the score, the more influential the variable is to the model). where a higher ROC represents higher importance. Logistic Regression had the best performance as a classification model and again revealed that both large pneumothorax and incomplete viral delivery were significant.


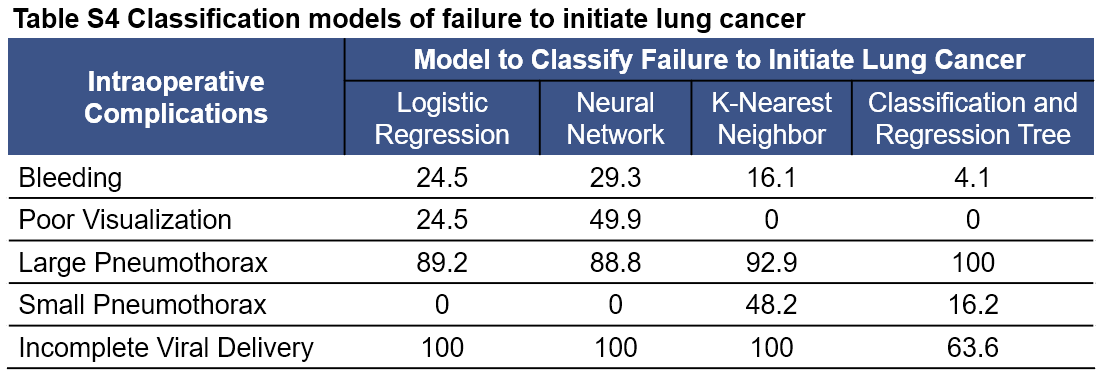


**(Table S5)** To validate the prediction power of only pneumothorax and incomplete delivery on failure to initiate lung cancer we determined Accuracy, AUC, Sensitivity/Specificity, Precision/F1 Score. Here, we again used 4 different machine learning models (logistic regression, neural network, K-nearest neighbor and classification/regression tree models) to determine which model had the best performance; logistic regression had the best performance.


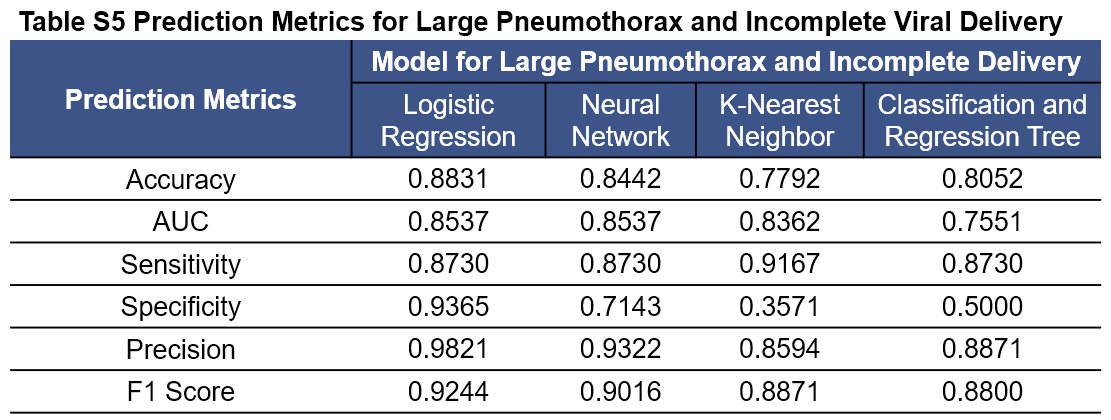


**Section S2. Results: Histological evaluation of primary and metastatic tumors consistent with lung adenocarcinoma**

To evaluate whether our model has histological tumor markers consistent with lung adenocarcinoma, we selected 3 mice as follows:

1. Normal Control male C57Bl/6 mouse with no cancer, lungs were harvested at the age of 6 months.
2. S6.KPP male mouse injected with Cre virus at the age of 3 months, lungs were harvested 3 months later at the age of 6 months
3. C6.KPP female mouse injected with Cre virus at the age of 3 months, lungs were harvested 3 months later at the age of 6 months

***Immunofluorescence of TTF-1: Fig. 2b (10X) and S1a (20X)*** TTF-1 is a transcription factor that regulates downstream genes including surfactant protein (A, B, C, and D), PD-L1, Mucin 1, and epithelial-mesenchymal transition markers (vimentin and E-cadherin). TTF-1 is predominantly expressed in alveolar type II cells (cover ~5% of the alveolar surface area) and Club cells (reside in smaller airways, bronchioles), but in the setting of cancer, can also be present in alveolar type I cells (cover ~95% of the alveolar surface area, responsible for oxygen diffusion therefore are in very close proximity to capillaries) and basal cells (reside in larger airways, trachea and bronchi). *Normal lung* TTF-1 expression is minimal and uniformly distributed along alveolar type II pneumocytes. *S6.KPP* In primary and metastatic tumors, TTF-1 expression is high, diffuse and extensive throughout the tumor regions. *C6.KPP* In primary and metastatic tumors, TTF-1 expression is high, but is more localized and concentrated within the tumor. Where TTF-1 (red) colocalizes with DAPI (blue, staining for nuclei), the overlap (purple) confirms that TTF-1 is nuclear. Areas appearing red only may be due to tissue thickness and imaging focus that is the three-dimensional nature of lung, or presence in larger airways. Yellow areas may indicate TTF-1 to be in close proximity to CD31 (green, marker of capillaries) such as alveolar type I cells.

***Immunofluorescence of NapA: Fig. 2c (10X) and S1b (20X)*** NapA is a protein that is involved in the maturation of surfactant proteins, particularly B and C, that in the setting of lung cancer, do not have an impaired function, but an increased function. *Normal lung* NapA expression is primarily associated with alveolar type II cells and is not known to be associated with club cells, alveolar type I cells or basal cells, thus it demonstrates a minimal and uniform distribution. *S6.KPP* Since Ad5SPC initiates tumors in alveolar type II cells, it is not surprising that S.KPP tumors overexpress NapA in a more diffuse infiltrative pattern. *C6.KPP* Interestingly in tumors initiated with Ad5CC10 Cre (Ad5CC10 virus initiates cancer in club cells that have the CC10), NapA is also overexpressed in a more concentrated and nodular pattern. This could be explained by the overexpression of TTF-1 in alveolar type I and club cells that may lead to genetic reprogramming resulting in expression of NapA. Where, NapA (red) may colocalize with nuclei of alveolar type II cells (blue) and appear purple, or NapA may be present in alveolar type I cells (that are in close proximity to CD31, green, marker of capillaries) and appear yellow.

***Immunofluorescence of PD-L1: Fig. 2d (10X) and S1c (20X)*** PD-L1 protein has a critical role in regulating immune responses and maintaining self-tolerance by binding to its receptor, PD-1, on the surface of T cells. Normal binding inhibits T cell activation and proliferation thereby reducing the immune response. However, in the setting of cancer, the upregulation of PD-l1 on the surface of tumor cells, allows it to evade immune surveillance. The use of immune checkpoint inhibitors blocks the binding of PD-L1 to PD-1 thereby reactivating exhausted T-cells and enhancing immune response against tumors. *Normal lung* Low level expression of PD-L1 is normal in alveolar type I and II cells, endothelial cells of blood vessels, and resident alveolar macrophages that collectively regulate immune homeostasis. *S6.KPP* Within the primary tumor, PD-L1 staining is again more diffuse; interestingly metastatic tumors show less intense PD-L1 staining compared to primary tumors. *C6.KPP* Primary tumors have a more intense PD-L1 staining compared to S6.KPP primary tumors; interestingly metastatic tumors have even more intense PD-L1 staining compared to primary tumors. Further quantitative scoring or genetic analyses would be needed to confirm these differences within metastatic lesions. Even though PD-L1 is on the cell membrane (or in the cytoplasm during transport and synthesis), tumors where PD-L1 (red) colocalizes with DAPI (blue, staining for nuclei) appearing purple may be cells where PD-L1 is in close proximity to nuclei.

**
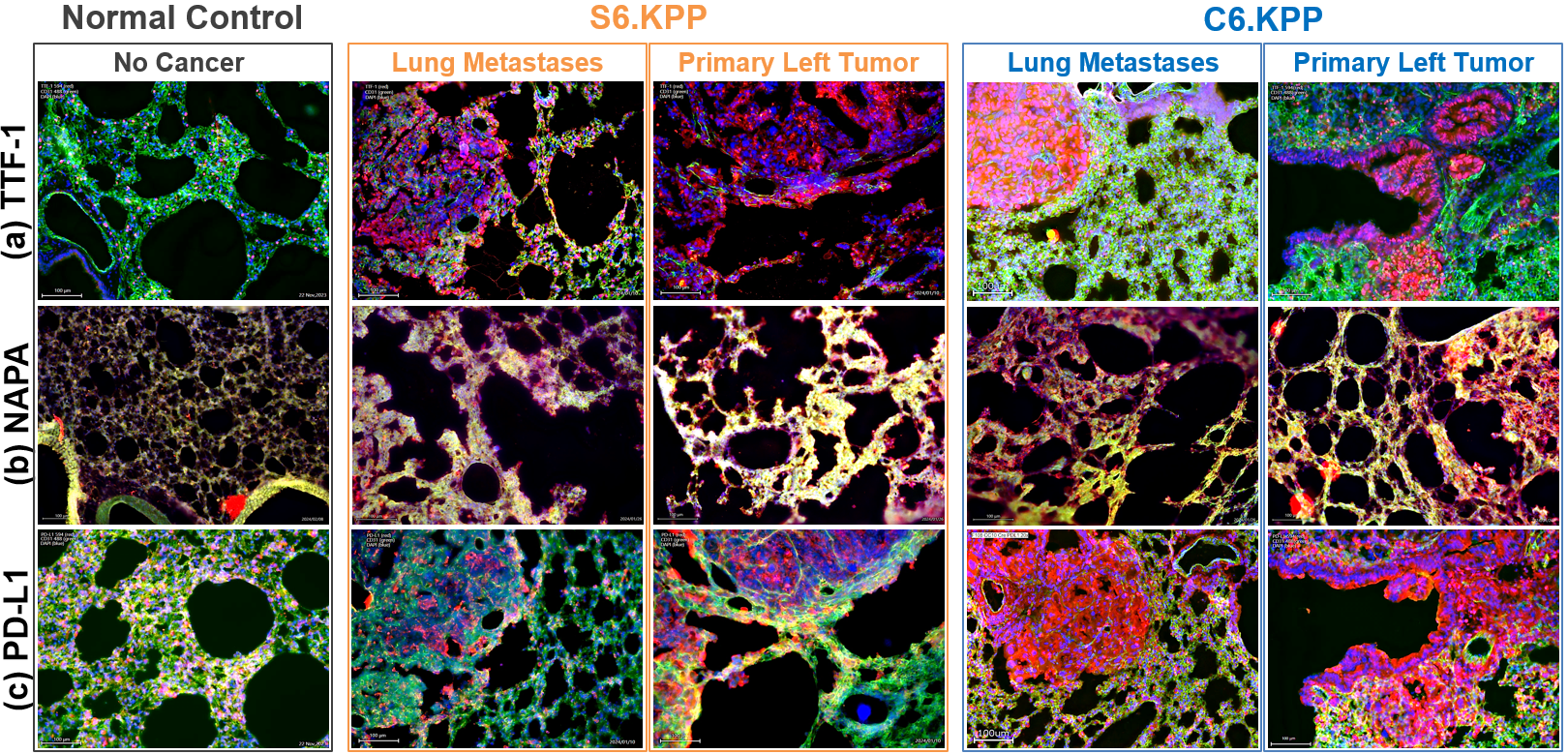
**

**Fig. S1 Histopathological analyses of Kras^G12D+/-^/p53^fl/fl^/myristoylated-p110α^fl/fl^-ROSA-gfp injected with either Ad5SPC or Ad5CC10 Cre virus at 20X magnification.** Representative IF 20X lung images from 10X images in Fig. 2. Again, Normal Control (grey box), S6.KPP (orange box, primary tumor on right, lung metastases on left), C6.KPP (blue box, primary tumor on right, lung metastases on left). In all images, CD31 is green, DAPI, 4,6-diamidino-2-phenylindole is blue, proteins of interest (a) TTF-1, (b) NAPA, (c) PD-L1 are red; scale bars=100um; for microscope settings, see Table S9. In the normal control lung with no cancer, there is minimal and uniformly distributed TTF-1, NapA, and PD-L1. In the S6.KPP model, there is a high expression of TTF-1, NapA, and PD-L1 that has a more diffuse and infiltrative pattern in the primary tumor and metastatic tumors. In contrast, C6.KPP models exhibit a more concentrated and nodular pattern of TTF-1, NapA, and PD-L1 in both the primary tumor and metastatic tumors, suggesting a more compact and concentrated tumor growth.

**Section S3. Results: Survival is a function of virus and concentration**

***Cohort 3 and 4: Cre and concentration specific survival:*** To analyze survival based on time of injection to death for S.KPP and C.KPP models at 3 different concentrations of virus, mice were pooled from experiments and included based on 2 criteria:

1. age-matched (3-4 months old at the time of injection)
2. did not undergo any uCT scans but were harvested at humane endpoints, specifically, >15% weight loss or hunching due to difficulty breathing

Mice did not undergo necropsy. We found that tumor initiation had the highest failure rate with the lowest concentration of virus, S5.KPP and C4.KPP, therefore they had the lowest numbers within the cohort.

***Cohort 3: S.KPP:*** total of 69 mice.

- S5.KPP had 13 mice (n=8 female and n=5 male)
- S6.KPP had 33 mice (n=17 female and n=16 male)
- S7.KPP had 23 mice (n=12 female and n=11 male)

***Cohort 4: C.KPP*:** total of 66 mice.

- C4.KPP had 13 mice (n=4 female and n=9 male)
- C5.KPP had 36 mice (n=20 female and n=16 male)
- C6.KPP had 17 mice (n=9 female and n=8 male)

**Section S4. Results: Metastasis frequency and location by virus and concentration**

***Cohort 5: S.KPP & C.KPP (additional details)*** To analyze frequency and location of metastases of both models at different concentrations of virus, mice were pooled from experiments and were included based on 2 criteria:

1. age-matched (3-4 months old at the time of injection)
2. available necropsy notes that confirmed **visual** macro metastases.

Please note we cannot report on metastases that are deep within the organ that could potentially be visualized with a whole body uCT scan or histological survey.

*S.KPP mice* divided as follows:

1. S5.KPP, 5 mice (4 female, 1 male)
2. S6.KPP, 9 mice (3 female, 6 male)
3. S7.KPP, 11 mice (5 female, 6 male)

*C.KPP mice* divided as follows:

1. C4.KPP, 5 mice (4 female, 1 male)
2. C5.KPP, 11 mice (6 female, 5 male)
3. C6.KPP, 18 mice (8 female, 10 male).

lung tumors had an ovoid shape that is solid and whitish appearance post-perfusion most likely due to outgrowing its blood supply. In contrast, normal lung tissue deflates upon opening the intrathoracic space due to absence of negative pressure and has reddish/pinkish appearance post-perfusion due to intact blood supply. Metastatic mediastinal lymph nodes (MLN) are not only large but solid compared to normal MLN which is stringy and difficult to visualize at necropsy. Metastases to the ribs appear as a clear tumor growing from the bone and metastases to the heart are also solid and appear to have outgrown its blood supply. Tumors on the diaphragm and malignant bloody pleural effusions can be visualized after opening the abdomen and before cutting the diaphragm to obtain access to the thorax. Spinal metastases appear as a clear tumor growing from the bone and are present in mice unable to move their hind legs. Metastases to the kidney and liver also have an ovoid shape that is solid and whitish compared to surrounding normal tissue.

***
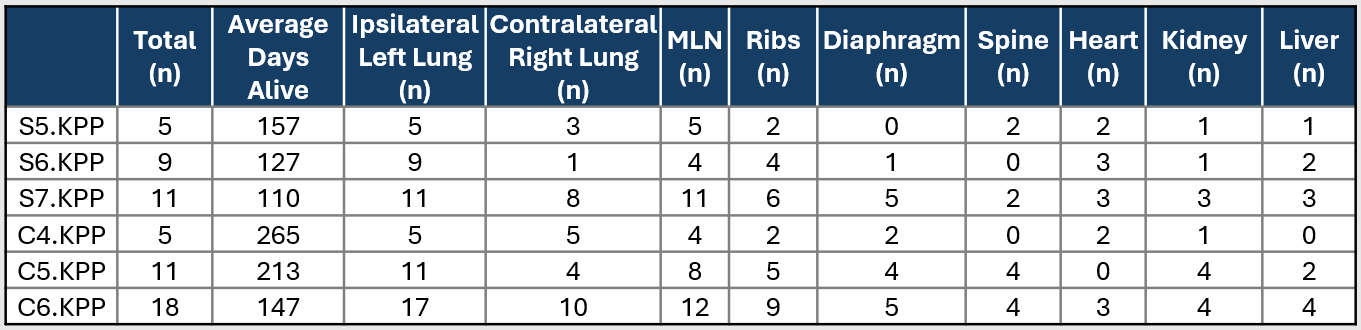
***

**Section S5. Results: Timing of cancer progression and metastasis recapitulates clinical lung cancer**

***Cohort 6: S.KPP (additional details)*** To evaluate timing of cancer progression and metastases, 12 S6.KPP mice were injected and included on 6 criteria:

1. age matched (3 months old at the time of injection)
2. gender-matched (6 female and 6 male)
3. Had no serious intraoperative complications during Cre virus injection
4. Initiated tumors at least 125 days post injection
5. Received continuing uCT scans after confirmation of cancer initiation to determine timing of first metastases
6. Received a final uCT scan performed the day before harvest based on humane endpoints.

Since we knew that tumors were not visible the first 4 weeks after Cre injections, the first uCT scan was at 4 weeks, then scans were performed weekly to note time of tumor initiation and time to first visualization of a metastatic tumor. For this data analysis, we defined metastases as either an ipsilateral metastatic (left) or contralateral (right) lung tumor.

**(Table S6)** Listed are each of the twelve S6.KPP mice including gender, date of birth, and date of injection with Ad5SPC. Results include days to:

1. identification of primary tumor on uCT scan
2. identification of first metastases (either ipsilateral or contralateral)
3. Death and specific Date.

Injections of both Male and Female groups were done on two separate dates, spread months apart (5/19/22 and 9/14/22), to highlight consistency in technique and consistency in the model to reproducibly produce primary tumors, metastasis, and Death in similar times.


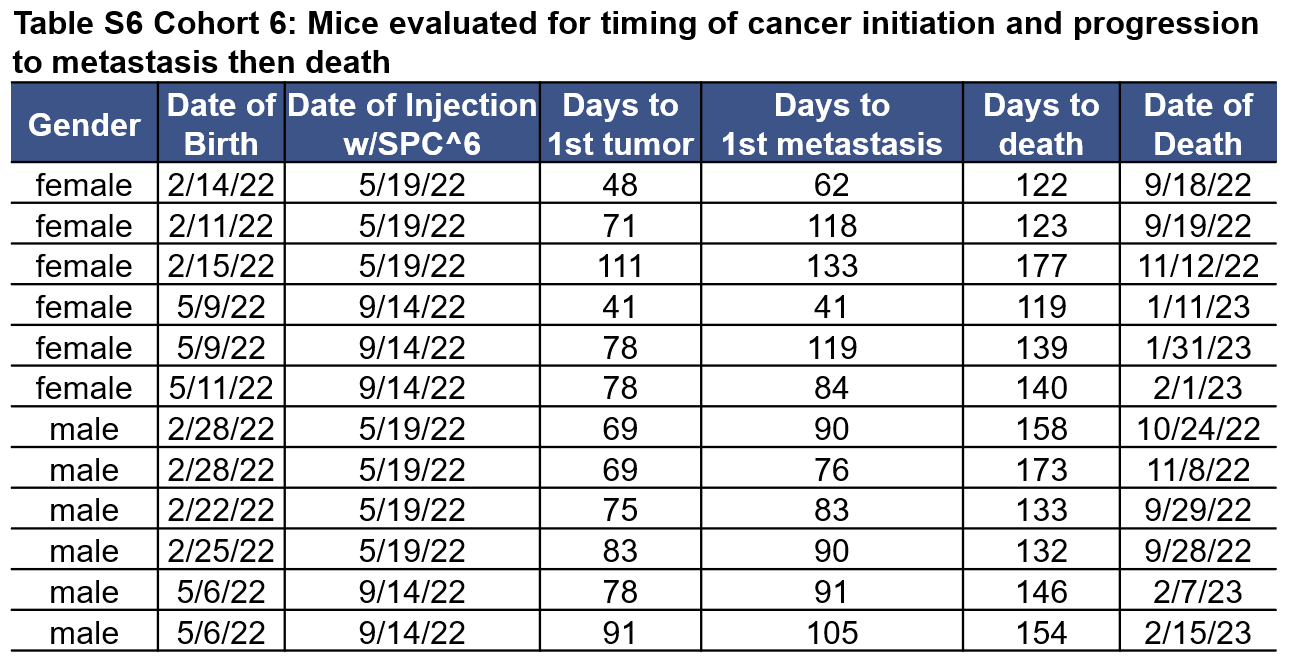


**Section S6. Results: Immune Profiles of Mice and Men display similar Lung cancer behavior**

***Cohort 7: S7.KPP & No Cancer (additional details)*** To demonstrate stage of cancer impacts the immune response which would impact the tumor microenvironment, mice were injected with Ad5SPC at a concentration of 10^7^ pfu when they were 3 months old. Based on trends within our data, we determined *(1) S7.KPP-Early* stage cancer to be a tumor volume of 3 to 8mm^3^ which was when mice were ~5-6 months old at time of harvest; *(2) S7.KPP-Late* stage cancer to be a tumor volume 50 to 85mm^3^ which was when mice were ~6-7 months old at time of harvest; *(3) No cancer* mice were ~4.5 to 6 months old at the time of harvest. Since we knew that tumors were not visible the first 4 weeks after Cre injections, the first uCT scan was at 4 weeks, then scans were performed weekly to note time of tumor initiation when tumor volume reached endpoint, mice were harvested 1-2 days after the uCT scan because we found that mice harvested the day of the uCT scan has inconsistent immune profiles.

In **Fig. S2**, we present comprehensive immune profile comparisons between S7.KPP-Early stage, S7.KPP-Late stage, and No cancer. For each mouse, we collected samples from six organs: Left lung (L.Lg), Right lung (R.Lg), Mediastinal Lymph Nodes (MLN), Thymus (THY), Spleen (SPL) and Bone Marrow (BM). We analyzed 100,000 cells from each organ to determine the following immune cell populations: Natural Killer (NK), Cytotoxic T lymphocytes (CD8+), regulatory T cells (Treg), polymorphonuclear myeloid-derived suppressor cells (PMDSC), monocytic myeloid-derived suppressor cells (MMDSC), M1 and M2 macrophages. We can make 3 general observations:

*Increased “killing” immune cells* Under non-cancer settings, CD8+ T cells have a critical role in identifying and destroying infected cells and can differentiate into memory T cells to provide long-lasting immunity. NK cells are part of the innate immune system, capable of killing target cells without prior sensitization. In cancer, both CD8+ and NK cells can recognize cancer cells and secrete cytokines to activate macrophages and enhance killing. Consistent with this, we observed increased levels of CD8+ T cells and NK cells in the Left Lung, Right Lung, Thymus, and Spleen in the S7.KPP-Early stage group, suggesting a stronger cytotoxic immune response in these organs. This increase was not observed in the S7.KPP-Late stage group.

*Increased “suppression” immune cells* In non-cancer settings, suppressive immune cells have a critical role in preventing autoimmunity. In cancer, regulatory T cells and myeloid-derived suppressor cells, including PMDSC and MMDSC can accumulate within the tumor microenvironment and facilitate tumor growth and immune evasion. Our data demonstrates a significant increase in suppressive immune cells in the S7.KPP-Late stage group compared to S7.KPP-Early stage group. Both cancer groups (S7.KPP-Early stage and S7.KPP-Late stage) exhibit higher levels of suppressive cells than the No cancer group, indicating increased immune suppression in the presence of cancer.

*Tumor Associated Macrophages (TAM)* Under non-cancer settings, M1 macrophages respond to infections and tissue damage, while M2 macrophages resolve inflammation and promote tissue repair and remodeling. In cancer, TAMs, derived from circulating monocytes, are predominantly more M2-like, supporting tumor growth and metastases. Our data show elevated levels of both M1 and M2 macrophages in the S7.KPP-Early stage group compared to the No cancer group, though this increase was not statistically significant in the S7.KPP-Late stage group. This suggests a complex role of TAMs in the tumor microenvironment, with potential shifts in macrophage polarization during cancer progression.

**
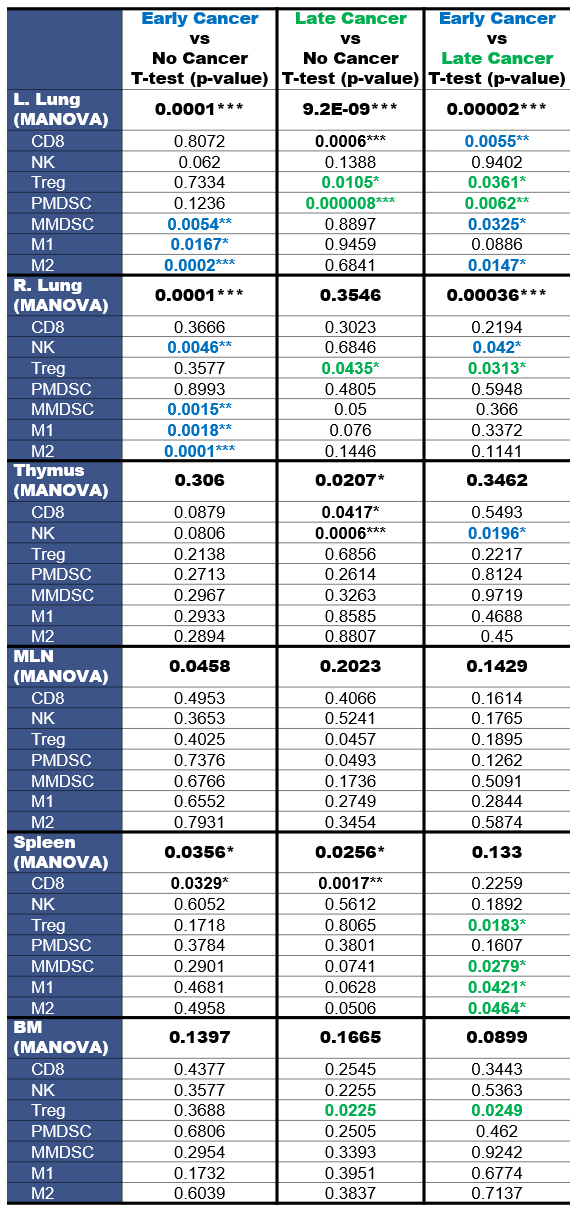
**

**Fig. S2 Immune profiles across six different organs comparing S7.KPP-Late vs S7.KPP-Early vs No cancer.** Statistical analyses (t-statistic, p-value) indicate significant differences in immune cell populations between different groups tested. p-Values of MANOVA of each organ site is shown to show significant aggregate differences of immune cells in each comparison. Significant increased populations of specific immune cells in each organ is highlighted in the same color of the group which is significantly increased. p-value < 0.05 = (*), p-value < 0.005 = (**), p-value < 0.001 = (***).

**Section S7. Results: Rate of Tumor Progression to Cisplatin and Immunotherapy May depend on initial size**

***Cohort 8: Response to Treatment by Starting Volume:*** For preliminary data of tumor responsiveness and how starting volume may effect response to treatment, S6.KPP and S7.KPP mice were combined and treated with either cisplatin or immunotherapy to evaluate whether they exhibit primary resistance, primary response/secondary resistance, compared to control mice (+cancer, no treatment). Mice were pooled from experiments and included based on 2 criteria:

1. age matched (3 months old at the time of injection)
2. obtained uCT scan to determine starting tumor volume before starting treatment and had serial weekly scans during treatment.
   1. Control mice were selected if they obtained weekly scans after confirmation of primary lung tumor, some mice were treated with 1XPBS and some were not; since there was no difference between controls, they were grouped together.

***Cohort 8: S6.KPP and S7.KPP (additional details of medium size start group, Immunotherapy Treatment)*** We grouped S6.KPP and S7.KPP mice together as there was no difference when assuming linear or log calculation of slopes. For the linear days analysis, the slopes were calculated as the change in values over time for each column. The calculated slopes for S6KPP were 8.13 for D33, 0.39 for E135, and 4.44 for E156. For S7KPP, the slopes were 0.45 for S177, 2.83 for S197, 1.29 for S209, and 0.33 for V5. A t-test was performed to compare the slopes between the two groups. The resulting t-statistic was 1.55, with a p-value of 0.181. This p-value indicates that there is no statistically significant difference between the trends of the S6KPP and S7KPP groups at the conventional significance level of 0.05, suggesting that the growth patterns of the two groups are statistically similar. For the log-transformed days analysis, the log of the days was taken, and the slopes were recalculated, now including the data for mouse E123. The calculated slopes for S6KPP were 199.86 for D33, -1.22 for E123, 9.76 for E135, and 110.90 for E156. For S7KPP, the slopes were 10.36 for S177, 67.77 for S197, 29.66 for S209, and 8.58 for V5. A t-test was again performed to compare the slopes between the two groups. The resulting t-statistic was 1.03, with a p-value of 0.343. This p-value also indicates that there is no statistically significant difference between the trends of the S6KPP and S7KPP groups at the conventional significance level of 0.05. Overall, both the linear and log-transformed analyses suggest that the trends of **medium size start groups**, Immunotherapy treatment between the S6KPP and S7KPP groups are statistically similar, with no significant difference in their growth patterns. **Table S7** lists all mice in S.KPP **medium size start** group.

***Cohort 8: S6.KPP and S7.KPP (additional details of large size start group, Immunotherapy Treatment)*** We grouped S6.KPP and S7.KPP mice together as there was no difference when assuming linear or log calculation of slopes. For the linear days analysis, the calculated slopes for S6KPP were 8.94 for D35, 5.30 for E130, and 5.29 for E155. For S7KPP, the slopes were 8.21 for S168, 5.99 for S185, 2.13 for S248, and 0.95 for S322. A t-test was performed to compare the slopes between the two groups, resulting in a t-statistic of 0.98 and a p-value of 0.37. This p-value indicates that there is no statistically significant difference between the trends of the S6KPP and S7KPP groups at the conventional significance level of 0.05, suggesting that the growth patterns of the two groups are statistically similar. For the log-transformed days analysis, the log of the days was taken, and the slopes were recalculated. The calculated slopes for S6KPP were 220.72 for D35, 131.12 for E130, and 128.68 for E155. For S7KPP, the slopes were 188.37 for S168, 110.02 for S185, 51.90 for S248, and 20.55 for S322. A t-test was again performed to compare the slopes between the two groups, resulting in a t-statistic of 1.34 and a p-value of 0.24. This p-value also indicates that there is no statistically significant difference between the trends of the S6KPP and S7KPP groups at the conventional significance level of 0.05. Overall, both the linear and log-transformed analyses suggest that the trends of **large size start group** Immunotherapy treatment between the S6KPP and S7KPP groups are statistically similar, with no significant difference in their growth patterns. **Table S7** lists all mice in S.KPP **large size start** group.

***Cohort 8: No treatment (additional details of multiple use)*** Since we had an insufficient number of control mice, some control mice were included in different starting size groups if they had a uCT scan within the parameters of the respective starting group size, which would then be considered day 0 starting treatment, as the mouse would never go on to receive a treatment. In **Table S7,** to provide transparency as to how many times one control mouse was used in early, middle and late stage, each mouse, identified by their Mouse ID, and denoted as “A” for its first inclusion, “B” for its second inclusion, and “C” for its third inclusion. Please note that we plotted each treated mouse once.

**Table S7 Cohort 8: Exact Mouse IDs included in each starting size group, categorized by starting volume and treatment.**


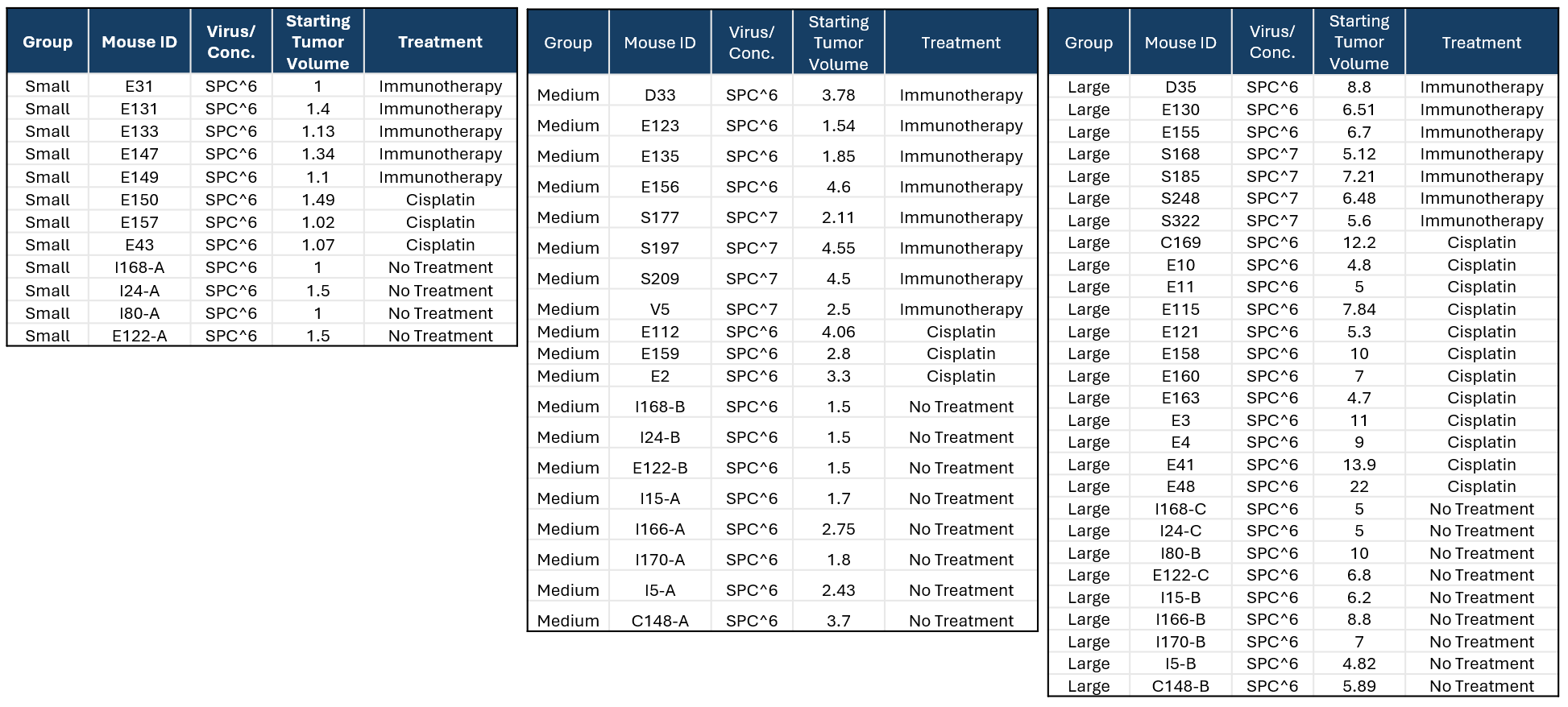


**Methods**

**Mouse Husbandry**

For breeding, one male, triple transgenic Kras^G12D+/-^ mouse was housed with two female, triple transgenic Kras^G12D-/-^ mice. After babies were born, sunflower seeds were given to promote lactation. When cages became overcrowded, but babies were not weaned, mice were moved to larger hamster-sized cages to provide adequate space for larger litter. Hamster-sized cages were maintained with identical bedding, food, and water sources. Mice were weaned at 21-24 days old, ear tagged, and tail clipped for genotyping at 6-8 weeks old using PCR. All mice in Cohorts 1-9, were homozygous for p53^fl/fl^ deletion and myristoylated p110α^fl/fl^ ROSA-gfp. Mice were housed by gender, age, and genotype into cages of 5; age range within 1 cage < 1 week apart. Living conditions were strictly monitored and remained consistent throughout the experimental period. They were provided a diet of water and rodent diet pellets. Cages were changed weekly by vivarium staff, and the mice were visually inspected daily. Any mice displaying signs of distress, such as improper grooming or severe weight loss (>15% total body weight) were promptly harvested and/or euthanized based on their experimental group assignments.

**Survival Surgery of Intrathoracic injection of Cre virus**

*Step 1: Surgical Field Preparation*

Person #1 deeply anesthetizes the mouse using continuously inhaled isoflurane mixed with O2 at a flow rate of 1.5L/min. When pedal reflexes are absent, a subcutaneous (SQ) injection of 0.05mg/kg buprenorphine and 5mg/kg meloxicam in the fat pad between the ears is administered and hind toenails are trimmed to prevent the mouse from scratching and reopening their incision post-operatively. The mouse is then positioned with their right side down such that the left lung is up. We specifically inject the left lung because it has one lobe, and it is easier to identify metastases. Landmarks for the surgical site is visualized: a longitudinal axis is drawn from the base of the left ear to the tail, and a latitudinal axis is perpendicular to the longitudinal axis below the left arm, such that the intersection coincides with the largest amplitude of breathing movement. The mouse is shaved at the intersection, ~1cm wide and ~2cm long using a 0.5mm shaver, after which the surgical site is sprayed with 70% isopropyl alcohol alternated with chlorhexidine and repeated 3 times. When ready to begin surgery, adjust the isoflurane mixer to keep respiratory rate as low as possible.

Person #2 is keeping surgical notes of Mouse ID including time of injection (relative to Cre virus preparation), complications of injection, getting the next mouse ready, monitoring previous mice post-operatively, drawing up the Cre virus. *Cre virus* Gently pipette the Cre virus solution up and down 5 times, then pulling up 10uL of Cre viral solution onto the lid of the Eppendorf tube. Using an insulin syringe that has a 29G 1/2” needle, draw up the 10uL of Cre virus solution, being very careful to not introduce air within the syringe as it will cause a large pneumothorax and/or incomplete viral delivery that significantly impacts cancer initiation; the insulin syringe is placed on the table near Person #1.

*Step 2: Incision and Intrathoracic Injection of Cre Virus*

Person #1 uses Adson dressing serrated forceps to make a small fold in the skin (Fig. 1a, i) and iris scissors are used to make an incision ~0.5 cm (folded) so it is ~1 cm unfolded. Blunt dissection with scissors is conducted to visualize the fat pad. Adson serrated far tooth forceps are used to pick up the fat pad (Fig. 1a, ii) and scissors release the fat pad along the bottom and sides until able to hold the flap of fat (Fig. 1a, iii). Once the lung is visualized (Fig. 1a, iv) hold the incision open with curved forceps, and identify site of injection: midaxillary line, ~2 ribs above the bottom edge of the lung, in between 2 ribs.

Person #1 will pick up the syringe and hold the needle tip at a 90° angle, perpendicular to the lung with the bevel up; carefully count 1-2 breaths of the mouse, and immediately after a breath inject the needle into the lung (Fig.. 1a, v), ~1mm more than the length of the entire bevel. If the bevel if not entirely within the lung, this will result in an incomplete delivery of virus. With the needle in the lung, after allowing the mouse to take 1-2 breaths, immediately after a breath, Person #2 will **slowly** push the plunger of the insulin syringe. After Person #2 releases the plunger, Person #1 will hold the needle in the lung for an additional ~3-4 breaths so fluid does not escape, then retract the needle out at the same angle it was injected.

*Step 3: Closing the incision*

Person #1 uses forceps to gently pull the fat pad down and cover the injection site (Fig. 1a, vi), being careful there are no stray hairs near the incision site. Using curved forceps in left hand and Adson dressing serrated forceps in right hand, approximate the skin edges of the incision so they touch (Fig. 1a, vii) and pull the length of the incision site in opposite directions (left hand toward ear and right hand toward tail). Once approximated, open the incision slightly and drop surgical glue across the entire length of the incision site (Fig. 1a, viii). Using silicon tips (we use the needle cap from the insulin syringe), gently approximate the incision site without touching the glue (Fig. 1a, ix). The glue is usually cured within 8 seconds, but we found that the incision site can still open. At this point, turn off the anesthesia, then manually approximate the incision closed using a gloved hand lightly sprayed with 70% isopropyl alcohol to prevent the glove from sticking to the surgical site and opening the wound. We then add a second layer of glue on top of the incision and again manually approximate the incision using a gloved hand sprayed with 70% isopropyl alcohol; this is repeated with a third layer of glue. The final result is a closed incision site (Fig. 1a, x).

*Step 4: Postoperative recovery*

Person #2 puts the mouse into a clean cage with the incision facing up, but the mouse is not supine. A red heat lamp that keeps ~2/3 of the cage warm and ~⅓ of the cage cool (not under a red heat lamp); this allows the mouse to be kept warm while coming out of anesthesia but has the liberty of move to a cooler location if it is too warm. With careful monitoring, typically the mouse will start to move within minutes and fully moving within 5 minutes. We keep all mice from one cage to recover together and Person #2 closely observes the mice until they all resume normal activity, including eating and drinking. For the next couple of days, incisions are inspected; if an incision is open, the mouse is placed under anesthesia and the wound inspected. If the wound is superficial, the incision is cleaned and reapproximated with surgical glue, given another dose of buprenorphine 0.5mg/kg XR with meloxicam 5mg/kg and separated from cage mates until the wound heals. If the wound is deep or has an abscess, the mouse is sacrificed.

Preventing wound dehiscence is a major non-lethal complication that can cause infection or a chronic wound that does not heal, which not only alters the TME and immune response, but in some instances require euthanasia. Initially, we had significant wound dehiscence but this was largely solved by (1) cutting the toenails under anesthesia so when the mouse scratched a healing wound, it would not reopen; (2) closing the wound with surgical glue for a total of 3 times; (3) removing hard-plastic enrichment devices in home cages for about one week to prevent sharp edges catching the acute wound and opening it.

We initially used a Hamilton syringe with the shortest bevel (a blunt edge will not get through the pleural linings) but found that the needle easily dulled after repeated use and contributed to a larger pneumothorax. We eventually chose to use insulin syringes because they not only have no hub (therefore no dead space), but also, they have a high gauge needle that leaves a very small pneumothorax and are widely available and inexpensive that keeps costs down and increases feasibility. Furthermore, to adapt our method to other GEM + Cre virus combinations, we recommend doing a full titration of virus concentration to optimize timing of metastases, survival and response to treatment.

**Serial Micro Computed Tomography (µCT) to Quantitate 3D Tumor Volumes**

Most mice underwent micro computed tomography (µCT) of the chest one month following survival surgery and typically every 1-2 weeks thereafter (there were times when mice could not be scanned due to issues out of our control). At Institution A, we utilized the Molecubes X-cube with the following settings: 50kV, 440uA, 480 exposures with 125 ms/exposure for a total of 48mGy per scan, reconstructed to 100um. At Institution B we used the Bruker Skyscan 1172 scanner (Bruker Corp., Billerica, MA) with the following settings: 50kV, 400uA, 180 exposures with 70ms/exposure for a total of 28mGy per scan, reconstructed to 35um.

For image acquisition, mice were anesthetized using continuously inhaled isoflurane at 1.5 L/min administered via nosecone. Within the scanner, mice were placed prone with a respiratory sensor pad under the abdomen and physiologic monitoring software employed for respiratory gating. Active scan time per mouse was largely under three minutes, radiation was localized to the chest only, allowing for more views with greater resolution and lower radiation. Images acquired from both scanners were analyzed using ITK snap, a freely available software [www.itksnap.org](http://www.itksnap.org/) to view and segment tumor volumes in the axial, sagittal, and coronal planes. Manual segmentation of the largest diameter in each plane was used to calculate an oval volume.

**Necropsy**

Mice were deeply sedated, and no pedal reflexes were observed to ensure humane and ethical practices. During the necropsy, the chest cavity was opened to confirm presence of primary tumor, lymph node size was classified in the mediastinum, mesenteric/paraspinal, and axillary regions, local and distal metastasis were recorded. All observations were documented and later transcribed to a comprehensive electronic database. The tissue designated for cell sorting was immediately dissected and placed in a 24 well plate filled with fresh 1×PBS. Tissue intended for H&E or immunofluorescence (IF) were collected only after the mouse was perfused, which involved administering 30 mL of 0.9% normal saline and then 30 mL of 4% formaldehyde (formalin).

**Immunofluorescence**

Mouse lungs will float when immersed in formalin for cryopreservation, resulting in incomplete preservation and tissue damage upon freezing. To solve this problem, we optimized the following method: (1) The trachea up to the level of the vocal cords was visualized then we used suture to tie a knot right below the larynx; (2) the trachea was released by making an incision above the larynx so the suture will not slide off; (3) then, we dissected the thymus, left and right lungs, mediastinal lymph nodes, and heart as a single unit and gently placed into a 50mL conical tube with 4% formaldehyde; (4) the suture was hung over the top of 50mL conical tube before screwing on the lid; (5) the conical tube was then inverted (**Fig. S3**) allowing for complete immersion of the tissue.

**
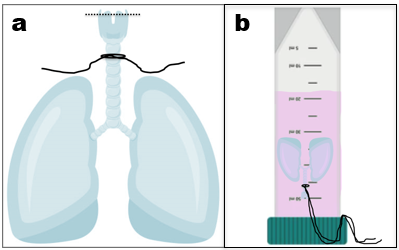
**

**Fig. S3 Harvesting the lung for immunohistochemistry.** (a) To prevent the suture from following off the trachea, we tied a knot below the larynx then made an incision above the larynx to release the trachea. (b) To completely immerse the lung in formalin, we draped the suture over the edge of the conical tube before screwing on the cap, then inverted the tube.

After 24 hours 4°C submerged in formalin, the tissue is placed in 30% sucrose for 48 hours at 4°C after which the tissues were placed in plastic cassettes with OCT (noting orientation) then covered complete with OCT. This was then frozen using methanol chilled with dry ice and stored at -80°C until ready to be sliced using the cryostat. Tissue sections 10 to 14 μm were sliced using a cryostat. To preserve lung architecture during immunohistochemistry protocol, adherent cryofilm (Section-lab, Hiroshima, Japan) was applied directly to the tissue then cryosliced. After slicing, the cryofilm with the tissue slice, was affixed to a microscope cover slip with CytoSeal 60 and dried overnight at 4 °C. The cover slip with cryofilm and tissue could be immediately used for H&E or immunofluorescence, or temporarily stored at −20 °C.

Tissue slices undergoing histopathologic analysis were rinsed with 1× PBS for 1–2 min ×3 to eliminate any residual OCT from the adherent side of the cryofilm, then air-dried for 1 h at room temperature. Considering the delicate nature of the tissue, we adopted a stainless-steel base mold (32 mm × 25 mm × 12 mm) as an alternative to traditional slide staining racks or dipping systems. For each step of the protocol, the cover slip with the tissue was placed inside the stainless-steel base mold and staining solution was added dropwise to the mold until the tissue was entirely immersed (approximately 400 µL) then placed into a humidifier, onto a rocker, at room temperature.

*Hematoxylin and Eosin* were processed following the manufacturer’s instructions (Vector Laboratories, Newark, CA, USA) with slight modifications. First, the cover slip with tissue was incubated with Hematoxylin for 5 min followed by two rinses with distilled water for 15 s each. To enhance contrast and facilitate nuclear staining, tissue was incubated in Bluing Reagent for 15 s followed by two rinses with distilled water for 15 s each; this optimized the visualization of cellular nuclei. The tissue was then dehydrated with increasing concentrations of ethanol (50%, 70%, and 100%), each step lasting 10 s. The tissue was then incubated with Eosin Y for 3 min to stain the cytoplasmic components followed by two rinses with 100% ethanol for 15 s each, then cleared with Xylene and air-dried at room temperature. All images in **Fig. 2** were obtained using white balance and automatic exposure (5 ms) setting.

*Immunofluorescence* staining, cover slip with the tissue on cryofilm was first permeabilized with a solution of 0.4% Triton/PBS for 20 min, followed by two rinses with 1× PBS for about 1–2 min each. We then blocked with 4% Normal Donkey Serum in 1×PBS for two hours, followed by two rinses with 1× PBS. Primary antibody concentrations were optimized in a solution of 1% bovine serum albumin and incubated at room temperature overnight (~16 h). The following morning, tissue was rinsed with 1× PBS for 15 min ×2 then incubated with secondary antibodies for two hours. Last, tissue was rinsed with 1× PBS for 15 min ×2 then incubated with DAPI for 10 min and rinsed again with 1× PBS for 15 min ×2. The cover slip is mounted onto a microscope slide and stored in an opaque slide case to avoid UV light exposure. All images in **Fig. 2** and **S1** were obtained with settings listed in **Table S9**. For immunofluorescence, the antibodies, concentrations and exposure times for micrographs for each condition are listed in **Table S9**.

**
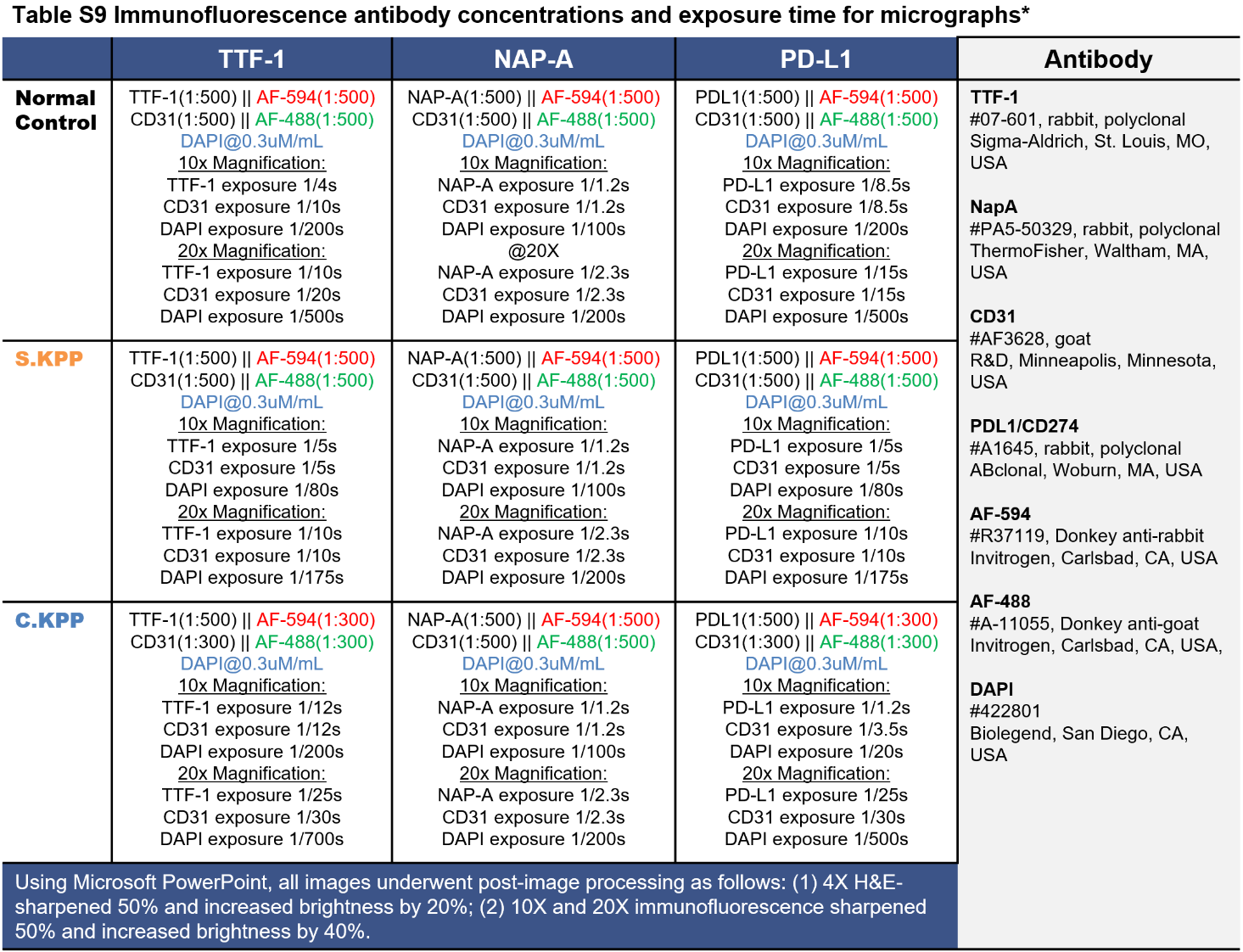
**

**Immune Profile**

The left lung, right lung, thymus, MLN, and spleen were separated, minced, and passed through a 40µm strainer (Falcon) using 1X PBS for single-cell suspension. Bone marrow was passed immediately through a 40um strainer; all samples were collected in Eppendorf tubes and spun down at 1000 x g for 5 mins. Samples pellets were then treated with red blood cell lysis buffer (1% ammonium oxalate), incubated for 5 mins at room temperature, spun down at 1000xg for 5 mins, and washed with 1mL 1X PBS. Sample pellets were resuspended with 1:300 Fc-block (Biolegend) in staining buffer (PBS containing 2% FBS and 1 mM EDTA). To assess viability, cells were stained with LIVE/DEAD Zombie aqua (1:500) according to the manufacturer’s protocol (Biolegend). Samples were then separated into 2 panels. In the first panel, live cells were stained for CD8+(CD45+,CD3+,CD8+), NK(CD45+,CD3-,CD49b+,NKG2D+), and Treg (CD45+,CD4+,CD25+,FoxP3+). For Tregs staining, cells were further fixed and permeabilized with the FOXP3 Intracellular Staining Kit (BioLegend). In the second panel, live cells were stained for P-MDSC(CD45+, CD11b+, Ly6C-hi, Ly6G-hi), TAM (CD45+, CD11b+, Ly6C-hi, Ly6G-lo), M-MDSC (TAM+, MHCII-lo), M1 (TAM+, F4/80-lo, MHCII+), M2 (TAM+, F4/80-lo, CD206+). Corresponding conjugated antibodies were diluted at 1:100 and incubated at room temperature for 30 mins in the dark.  Flow analysis was done using the CytoFLEX-S Flow Cytometer equipped with 3 lasers (405 nm violet, 488 nm blue, and 638 nm red; Beckman) and further analyzed with CytExpert analysis software. Antibodies used for immune profiles, as well as catalog numbers are included in **Table S10.**


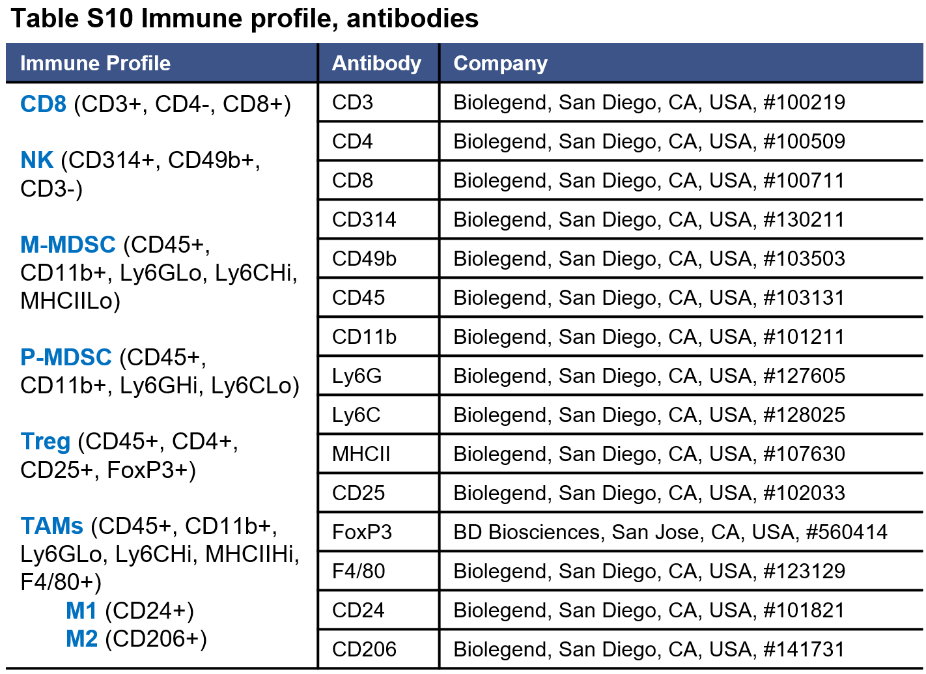

Supplement: Supplementary file 1 — Supplementary Information. [file 41598_2025_90344_MOESM1_ESM.docx]
